# Cadherin-26 (CDH26) regulates airway epithelial cell cytoskeletal structure and polarity

**DOI:** 10.1038/s41421-017-0006-x

**Published:** 2018-02-13

**Authors:** Marrah E. Lachowicz-Scroggins, Erin D. Gordon, Agata Wesolowska-Andersen, Nathan D. Jackson, Hannah J. MacLeod, Louis Z. Sharp, Matthew Sun, Max A. Seibold, John V. Fahy

**Affiliations:** 1 Cardiovascular Research Institute, University of California, San Francisco, San Francisco, CA 94143 USA; 2 Division of Pulmonary and Critical Care Medicine, University of California, San Francisco, San Francisco, CA 94143 USA; 3 Center for Genes, Environment, and Health, National Jewish Health, Denver, CO 80206 USA; 4 Johns Hopkins Bloomberg School of Public Health, W. Harry Feinstone Department of Molecular Microbiology and Immunology, Baltimore, MD 21205 USA; 5 Division of Pulmonary Sciences and Critical Care Medicine, Department of Medicine, Anschutz Medical Campus, University of Colorado, Aurora, CO 80045 USA

## Abstract

Polarization of the airway epithelial cells (AECs) in the airway lumen is critical to the proper function of the mucociliary escalator and maintenance of lung health, but the cellular requirements for polarization of AECs are poorly understood. Using human AECs and cell lines, we demonstrate that cadherin-26 (CDH26) is abundantly expressed in differentiated AECs, localizes to the cell apices near ciliary membranes, and has functional cadherin domains with homotypic binding. We find a unique and non-redundant role for CDH26, previously uncharacterized in AECs, in regulation of cell–cell contact and cell integrity through maintaining cytoskeletal structures. Overexpression of CDH26 in cells with a fibroblastoid phenotype increases contact inhibition and promotes monolayer formation and cortical actin structures. CDH26 expression is also important for localization of planar cell polarity proteins. Knockdown of CDH26 in AECs results in loss of cortical actin and disruption of CRB3 and other proteins associated with apical polarity. Together, our findings uncover previously unrecognized functions for CDH26 in the maintenance of actin cytoskeleton and apicobasal polarity of AECs.

## Introduction

Airway epithelial cells (AECs) create a physical barrier to inhaled particles and pathogens, regulate airway surface fluid, secrete mediators to recruit immune cells in response to injury, and help regulate smooth muscle cells to facilitate respiration^1^. To perform these functions, AECs form a complex and highly organized tissue with planar cell polarity, a differentiation process where cells organize with distinct apicobasolateral membranes to form ciliated epithelial cell sheets^2^. Basal progenitor cells in the airway epithelium serve as progenitor cells for different subtypes of epithelial cells (secretory, mucus and ciliated cells)^3^. Basal cells exhibit a pattern of polarity in their organization of proteins such as KRT14 and KRT5^4^ suggesting that formation of apicobasal domains happens early in formation of AEC sheets.

Actin interacts with multiple protein partners in ciliated epithelial cells to achieve the optimal cytoskeletal arrangements for the function of these cells^5, 6^. Several proteins in the apical tight junctions and in the basolateral adherens junctions play important roles in barrier function and polarization of AECs^7, 8^, but many details remain unknown. Cadherins are transmembrane proteins that facilitate actin reorganization and formation of epithelial cell sheets by mediating cell–cell adhesion^9^. The interaction between cadherin domains and their binding partners allows differentiating epithelial cells to change their shape and size and to form cell layers^10^. The cadherin superfamily is comprised of many proteins with different structures and functions, including classical cadherins, protocadherins, and atypical cadherins. Atypical cadherins such as FAT1^11^ and flamingo^12^ have atypical cytoplasmic domains that do not bind classical cadherin binding partners such as β-catenin, α-e-catenin, and p120/δ-1-catenin^13^.

Cadherin-26 (CDH26) is an atypical cadherin expressed on human chromosome 20q13.33. The locus for *CDH26* has 23 exons that are variably spliced to generate multiple *CDH26* transcript variants, two of which are expressed in AECs^14–16^. CDH26 appears in lists of genes in AECs that are differentially expressed in asthma^17^, and it is also differentially expressed in esophageal epithelial cells in patients with eosinophilic esophagitis^18–20^. Despite the data that CDH26 is expressed in epithelial cells and associates with diseases of epithelial cell dysfunction, the function of CDH26 in AECs is unknown.

We set out here to explore the role of CDH26 in the cytoskeletal dynamics of AECs and in planar cell polarity. We specifically explored whether CDH26 has functional cadherin domains that regulate the actin cytoskeleton and the apicobasal polarity of AECs.

## Results

### CDH26A is highly expressed in AECs and localizes to the apical membrane

**Fig. 1:**
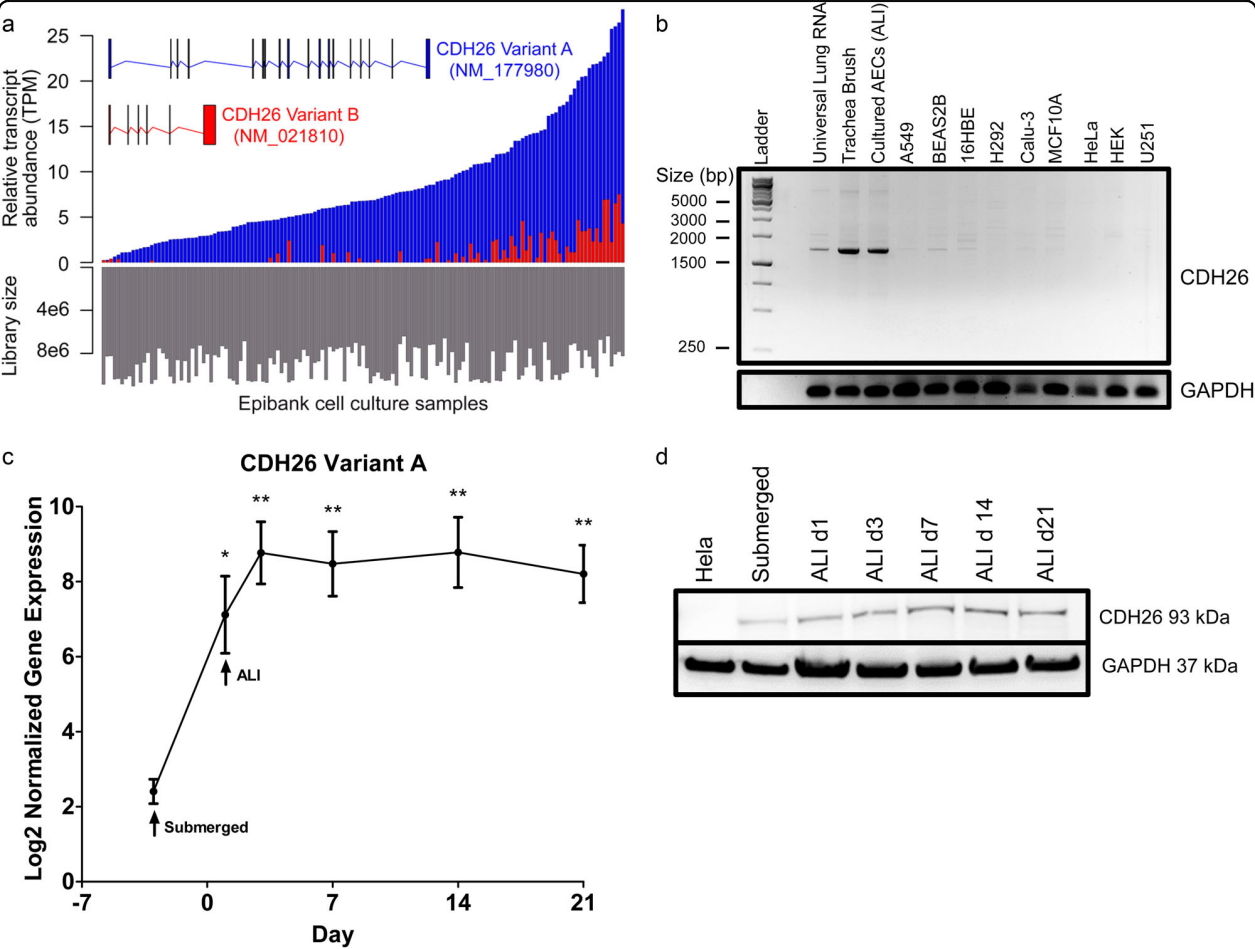
Cadherin-26 (CDH26) transcript variant A is expressed in bronchial epithelial cells. **a** Exon maps and patterns of gene expression for CDH26 variant A (blue) and variant B (red). Top plot: stacked bar plot of relative transcript abundance (in TPM = transcripts per million) for two CDH26 isoforms based on RNA-seq data for Epibank AECs (*n* = 141) grown at air–liquid interface (ALI) for at least 3 weeks. Bottom plot: bar plot of total library size (i.e., transcript abundances summed across all transcripts within a sample) for all AECs, arranged in the same order as the upper plot, demonstrating a lack of correspondence between increasing CDH26 expression and size of the dataset. **b** CDH26A transcript levels in a DNA gel for universal lung, fresh cell isolates from tracheas, AECs cells grown in culture, and clonal cell lines. GAPDH is use as the  PCR reaction loading control. **c** Time-dependent gene expression (means and s.e.m.) for CDH26A in EpiBank AECs (*n* = 5 donors) grown under submerged and ALI conditions. Asterisks indicate significant difference from submerged cells: *p < 0.05, **p < 0.005 (Kruskal–Wallis test with Dunn’s Multiple Comparisons test). Time points correspond to 1, 3, 7, 14, and 21 days at ALI. **d** Time-dependent protein expression for CDH26A in a representative donor grown under ALI conditions. HeLa cell lysate loaded as a negative control for lack of CDH26A protein expression  and GAPDH used as a protein loading control.

Two CDH26 transcripts are predicted from sequencing analysis of chromosome 20^21^, NM_177980 transcript variant A and NM_021810 transcript variant B. We explored the relative expression of these two isoforms in human AECs. CDH26 variant A has 3192 base pairs, 18 exons, and a predicted protein molecular weight of 92.4 kDa, whereas CDH26 variant B has 1092 base pairs, six exons, and a predicted protein molecular weight of 17.7 kDa (Fig. 1a, Supplementary Figure S1). I-TASSER 3D and PROSITE protein modeling of variant A predicts four cadherin domains, a transmembrane region, and a cytoplasmic domain^22, 23^. Modeling of CDH26 variant B with an alternative start exon shows a similar structure to the cytoplasmic domain predicted of variant A missing a transmembrane region.

To determine the relative expression of variant A and variant B in AECs, we generated whole transcriptome libraries from human AECs harvested from the tracheas of 141 cadaveric donors. This large biobank of AECs (‘Epibank’) has recently been described by us^24^. AECs in the Epibank were cultured at air–liquid interface (ALI) for at least three weeks to ensure full differentiation, after which RNA was extracted and cDNA libraries were sequenced as single end reads. The relative abundance of the two canonical CDH26 isoforms in each of the samples was measured in units of transcripts per million (TPM) using kallisto^25^, and plotted as stacked bar plots (Fig. 1a). These data showed that while expression of the two variants is strongly correlated (Spearman’s* ρ* = 0.61; *p*-value = 1.08 × 10^−15^), *CDH26* variant A is much more commonly expressed than *CDH26* variant B, with variant A representing an average of 95% of the *CDH26* TPMs across samples. Variation of *CDH26* expression across epithelial cell donors is explained in part by variation in library size (Spearman’s *ρ* = −0.19; *p* = 0.02). Because of its predominance in these samples, we focused further functional studies on *CDH26* variant A (hereafter referred to as CDH26A).

Using isoform-specific primers, we explored the mRNA levels of *CDH26A* in epithelial cell lines from different organs, in fresh cells harvested from human cadaver tracheas and in Epibank AECs cultured in submerged conditions, where cells were plated in media submerged for 24 h and then media removed taking the cells to ALI. Using end point PCR to amplify a region specific to CDH26A corresponding to exons 1–8 (Supplementary Figure S1B), we found expression of *CDH26A* in pooled human lung RNA, fresh trachea tissue and in AECs in culture (Fig. 1b). In contrast, we found little to no expression of *CDH26A* in 5 different lung epithelial cell lines (16HBE, BEAS2B, Calu-3, A549, H292) or in epithelial cells from kidney (HEK), breast (MCF10A), cervix (HeLa) or a non-epithelial cell glioblastoma cell line (U251), all of which lack the ability to fully differentiate due to having qualities of stemness^26–28^. Using qPCR in a time course of differentiation in five AECs donors, we found lower expression in cells cultured in submerged conditions and expression increased in cell cultures as early as 1 day at ALI, with a modest additional increase in well-differentiated cells at 21 days (Fig. 1c). We find protein expression for CDH26A to mirror our qPCR findings at ALI and to be  absent in the lysate of HeLa cells, selected as a negative control for antibody specificity (Fig. 1d).

**Fig. 2:**
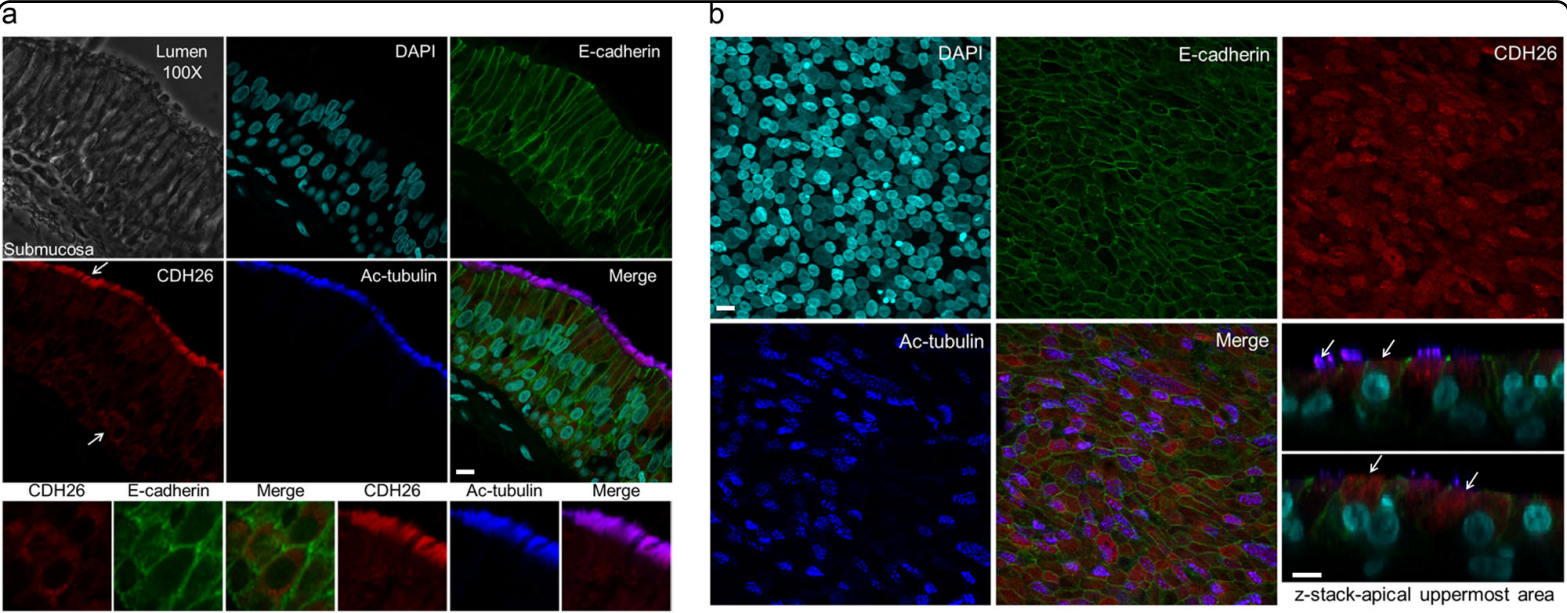
Cadherin-26 (CDH26) localizes to the apical membrane. **a** Immunofluorescence for E-cadherin (green), CDH26 (red), acetylated tubulin (Ac-tubulin, blue) and nuclei (aqua) in fresh tissue samples obtained from human tracheas. Bottom panels of A (indicated by white arrows):  zoomed into regions showing that CDH26 is non-junctional and predominantly in the apical membrane of cells. **b** Localization of CDH26A is maintained in AECs grown at ALI for at least 3 week in culture. Images are from a single donor and representative of the localization pattern of CDH26 from a survey of four different donors. Z-stack in B:zoomed region showing apical localization in cultured AECs, regions indicated by white arrows. Scale bar = 10 µm.

Using an antibody specific for the CDH26A isoform to probe AECs in airway mucosal tissue sections from human tracheal mucosa, we find that CDH26 immunolocalizes to apical regions of ciliated and goblet cells (Fig. 2a). Notably, CDH26A did not co-localize with E-cadherin at the junctions or with other junctional proteins such as alpha-e-catenin, beta-catenin or delta-1-catenin (Supplementary Figure S2). In AECs cultured at ALI, we also find that CDH26A also localizes to apical regions of the cells, mirroring the localization pattern we found in the native tracheal mucosa (Fig. 2b).

### CDH26A has functional cadherin domains and is involved in cytoskeletal dynamics

**Fig. 3:**
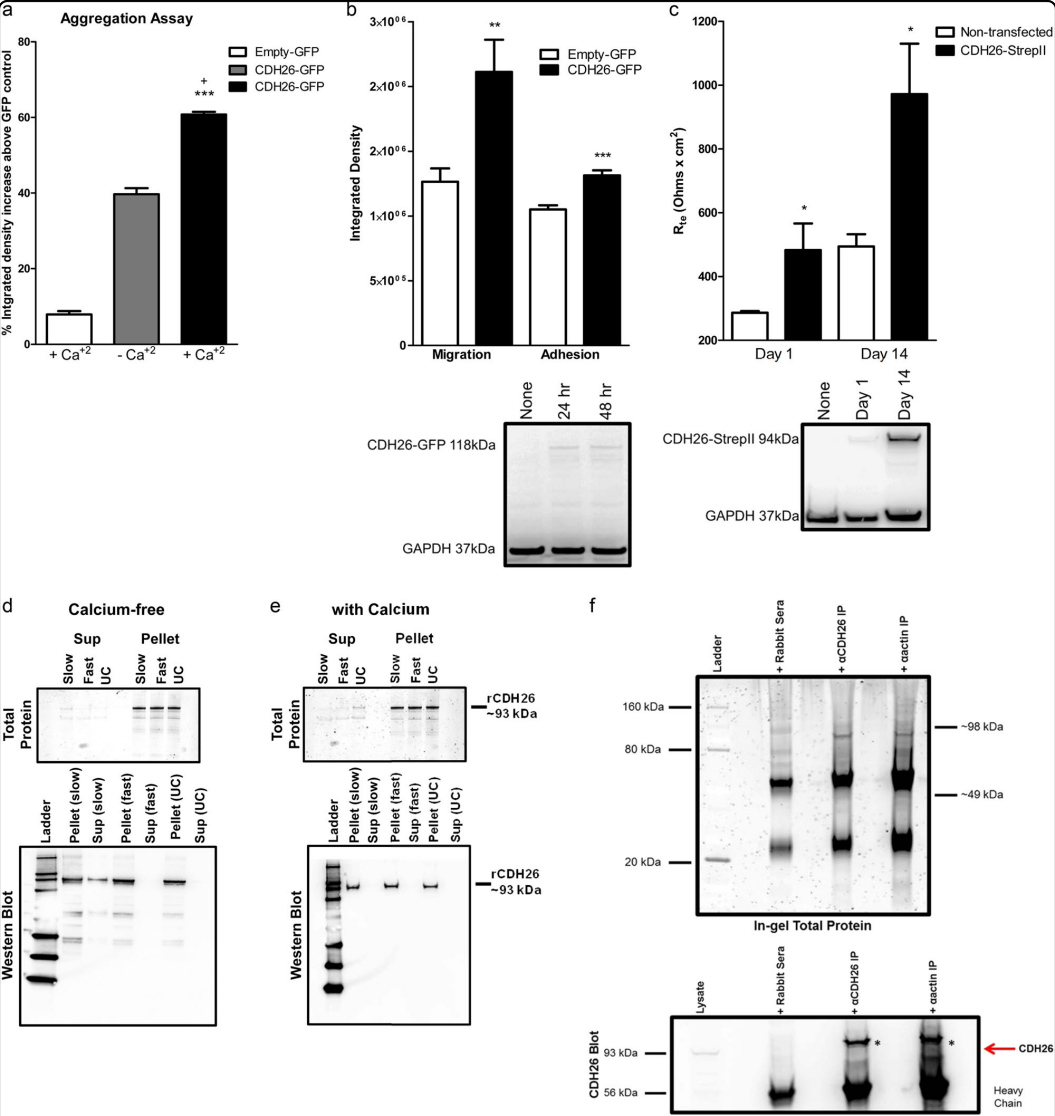
CDH26 variant A has functional cadherin domains. **a** Aggregation assay of CHO-K1 cells expressing Empty-GFP or CDH26-GFP in the presence or absence of calcium to test homotypic binding. Data are from 4 separate experiments with two replicates per experiment. Data presented as mean and s.e.m. *** indicates significantly different compared to empty-GFP CHO-K1 cells, *p* < 0.0001; ^+^indicates significantly different compared to CDH26-GFP without calcium, *p* < 0.05. **b** Migration and adhesion assays of HeLa cells expressing Empty-GFP or CDH26-GFP. Data are from *n* = 4 separate experiments with three replicates per experiment. Data presented as mean and s.e.m., statistics pair *t*-test where **p < 0.005 and ***p < 0.0001 is significant to Empty-GFP HeLa cells. Bottom panel—10µg of HeLa cell lysate from Empty-GFP or CDH26-GFP cells blotted with anti-CDH26 to verify expression of CDH26-GFP. GAPDH used as a protein  loading control. (**c**) Transepithelial resistance (TEER) measurements from untransfected HeLa cells or HeLa cells expressing CDH26-StrepII tag. *n* = 3 replicates and data presented as mean and s.e.m., statistics pair *t*-test where *p < 0.05 is significant to non-transfected HeLa cells. Bottom panel—10 µg of HeLa cell lysate from non-transfected or CDH26-StrepII cells blotted with anti-StrepII to verify expression of CDH26-StrepII. GAPDH used as a protein loading control. Recombinant CDH26 sedimentation assay in the absence of calcium (**d**) and in the presence of calcium (**e**) showing formation of multimeric protein aggregate structures. Top panels: in-gel staining for total protein. Bottom panels: blotted with anti-CDH26 to demonstrate sedimentation in each fraction. **f** Co-immunoprecipitation of CDH26 by actin from AECs visualized by in-gel total protein (upper) and western blot (lower) *Asterisks on the blot represent the IP bands pulled out in the co-IP reaction. Figure is representative of data obtained from *n* = 5 donor lysates from cells grown at ALI for at least 3 week in culture.

Type I classical cadherins exhibit homotypic binding through calcium-mediated interactions via their cadherin domains^29^. To test whether the cadherin domains in CDH26A are functional, we overexpressed full-length CDH26A protein with turboGFP tag in CHO-K1 cells. CHO-K1 cells maintain suspension-like cultures in the absence of extracellular matrix proteins^30^, and they are ideally suited to test whether CDH26A mediates cell aggregation via cadherin domains. We found that CDH26A markedly increased cell aggregation in CHO-K1 cells and that calcium further promoted aggregation (Fig. 3a). Thus, as full-length CDH26A caused cell aggregation we believe this is facilitated by calcium-dependent interactions between homotypic cadherin domains on the full-length protein. To explore whether the CDH26A cadherin domains facilitated cell movement and cell adhesion, we overexpressed CDH26A in HeLa cells using a variety of overexpression constructs. HeLa cells do not normally express CDH26A and so we used them as a model to study CDH26A function. Using an overexpression vector with a c-terminal GFP fusion protein to CDH26A, we found that HeLa-CDH26A cells showed increased migration in a Boyden chamber (transwell) assay (Fig. 3b). We also find increased adhesion in a matrix-binding assay by HeLa-CDH26A cells (Fig. 3b).

To determine whether CDH26A has a role in barrier function through binding interactions via its cadherin domains, we measured transepithelial electrical resistance (TEER) on HeLa cells grown on porous inserts. TEER is measured as current passes both through transcellular and paracellular paths. The transcellular resistance is made by the apicobasal membranes and paracellular resistance results from cell–substrate and cell–cell contacts^31^. Baseline TEER of HeLa cells was compared to that of cells with an overexpression vector containing a small StrepII tag fused to CDH26A in order to express full-length protein. We found that HeLa-CDH26A/StrepII cells had marked and stable increases in transepithelial resistance (Fig. 3c). Because TEER increases with monolayer formation via contact inhibition as cells make cell–cell contacts^32^, the increased TEER in HeLa-CDH26A cells expressing full-length CDH26A suggests an increase in adhesion by cell–cell or cell–substrate contact facilitated by homotypic binding via cadherin domains in the protein.

To further explore the functionality of CDH26A cadherin domains, we synthesized full-length recombinant CDH26A (rCDH26A) and found that the multimeric protein was present in cell lysates after analytical size exclusion chromatography (Supplementary Figure S3), in pellet fractions after centrifugation and was maximized by ultracentrifugation (Fig. 3d, e). Although the sedimentation equilibrium rate was not calculated for rCDH26A, we find that rCDH26A protein was present in pellet fractions after centrifugation with calcium during ultracentrifugation at 100,000 × *g* and to a lesser degree at a lower centrifugation speed of 5000 × *g*. As the predicted binding interaction on CDH26A is via multiple homotypic cadherin domains, pelleting recombinant CDH26A with or without calcium indicates that the formation of macromolecules is likely mediated by interactions of these calcium-sensitive cadherin domains (Fig. 3d).

To determine whether the aggregation, cell movement, adhesion, and increase in TEER is a direct or indirect effect of CDH26A binding to actin, we performed pull-down assay between CDH26A and actin. In both pull-down settings, we observed two bands in-gel with total protein stain corresponding to the molecular weight of CDH26A and actin (Fig. 3f, upper). In western blots we found that CDH26A co-immunoprecipitated with actin in both CDH26A and actin pull-downs (Fig. 3f, lower), but we were unable to observe any co-immunoprecipitation of other actin-binding partners such as p120/δ-1-catenin, β-catenin, or α-e-catenin in CDH26A pull-downs (data not shown). A limitation of these experiments is that they do not allow us to rule out that some other intermediary binding partner facilitates binding between CDH26A and actin.

**Fig. 4:**
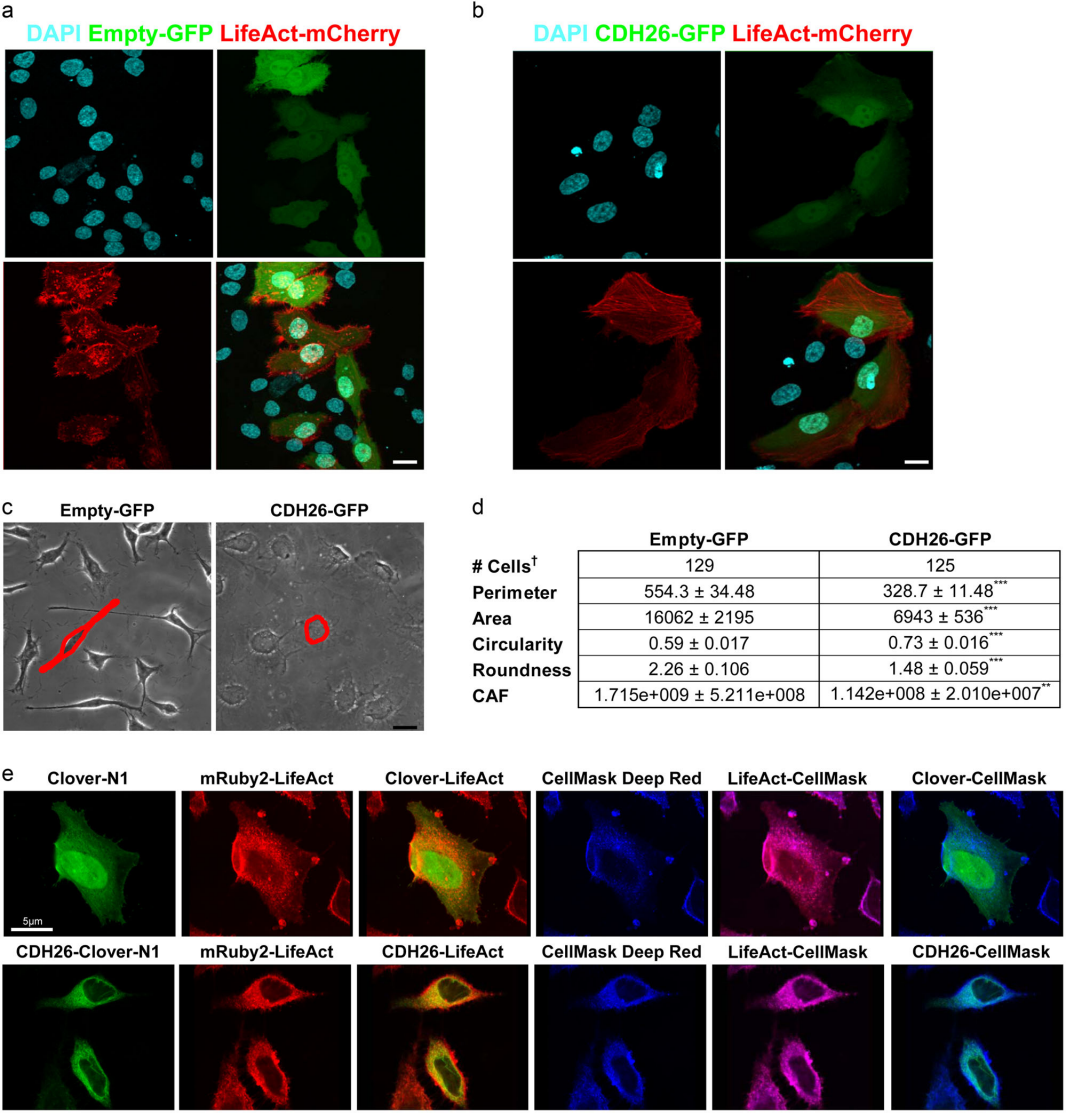
CDH26 variant A is involved in cytoskeletal dynamics. **a** HeLa cells co-expressing Empty-GFP and actin marker LifeAct mCherry show typical actin features of fibroblastoid cells. **b** Cells expressing CDH26-GFP have cortical actin, a feature of epithelioid cells, showing actin reorganization. Figures are representative of data from *n* = 3 separate experiments and four fields per condition. **c** Phase image for comparison of cell shape between Empty-GFP and CDH26-GFP cells—single-cell (red) selected to show shape difference between conditions. **d** Morphometric measurements made on stable expression in selected HeLa cells. ***p < 0.0001 and **p = 0.0028 significance, Unpaired *t*-test. ^†^Data from single cells measured, *n* = 14 fields at 60×, two separate experiments. **e** Super-resolution confocal microscopy images showing localization of Clover-N1 and CDH26-Clover-N1 (green) to mRuby2-LifeAct (red) and CellMask Deep Red (blue). Scale bars = 10 µm, unless otherwise noted.

**Fig. 5:**
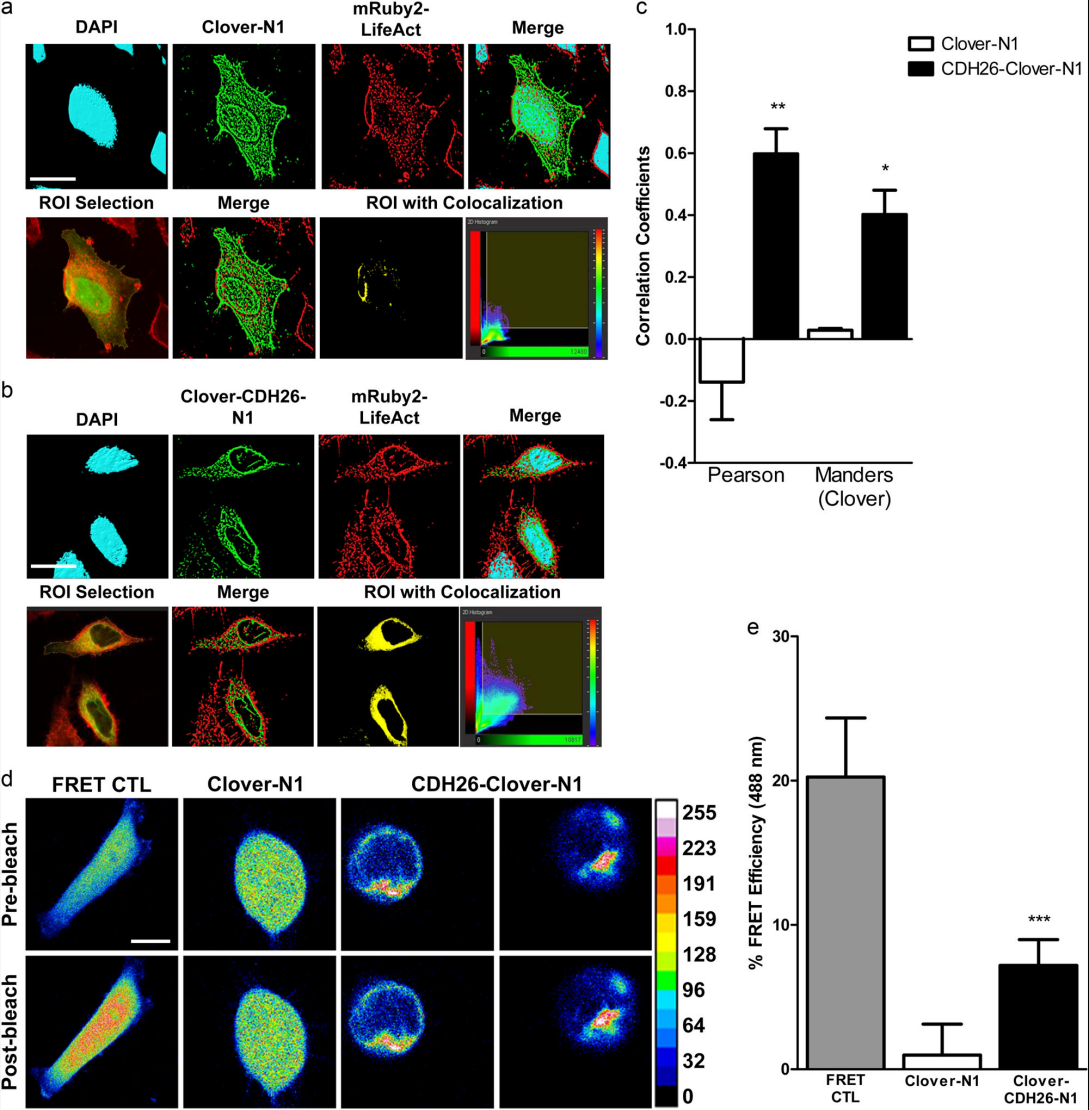
CDH26 variant A co-localizes with the actin cytoskeleton. **a** HeLa cells co-expressing Clover-N1 and actin marker mRuby2-LifeAct. **b** HeLa cells co-expressing CDH26-Clover-N1 and actin marker mRuby2-LifeAct show a co-localization pattern corresponding to cortical actin structures (yellow). Imaris 3D rendering to show relationships between fluorophors. ROI shows field selected to set threshold for co-localization. **c** Pearson’s correlation and Mander’s coefficient for the co-localization of Clover-mRuby2. Figures are representative of data from *n* = 3 separate experiments and 6 fields per condition. **d** Representative figure of the fluorescent intensities of the donor Clover presented as pre/post-photobleaching of the mRuby2 acceptor. Scale bar = 5 µm. **e** FRET efficiency measured in cells expressing Clover-N1 or CDH26-Clover-N1 as the donor and mRuby2-LifeAct as the acceptor fluorophore. Clover-10-mRuby2 as a positive control for FRET. Data from single cells measured, *n* = 8–10 fields at 60×, two separate experiments.

HeLa cells are fibroblast-like with increased motility, decreased contact inhibition and structures such as filopodia/puncta that are not present in AECs^33^. HeLa cells also have increased stress fibers indicative of cell spreading^34^, whereas AECs have highly organized cortical actin^35^. We wanted to visualize the effect of overexpression of CDH26A on cell shape and actin cytoskeleton in HeLa cells. To do this, we co-expressed an actin reporter (mCherry-LifeAct)^36^ and either empty-GFP or CDH26A-GFP in HeLa cells. We found that the HeLa-empty-GFP/LifeAct cells had a “fibroblastoid” phenotype without well-ordered cytoskeleton or actin structures (Fig. 4a, c). In contrast, HeLa-CDH26A/LifeAct cells formed cortical actin typical of epithelial cells (Fig. 4b). Single-cell morphometric measurements were made to determine whether overexpression of CDH26A resulted in formation of cortical actin. Here, circularity refers to the morphology of the cells in relation to the formation of cortical actin structures. In general, an epithelial cell assumes a circular morphology in culture, while fibroblast-like cells are highly elongated. For circularity, a value of 1.0 indicates a perfect circle. For roundness, 1.0 indicates a perfect circle and larger values indicate oblong and non-circular objects^37^. A cell area factor (CAF) was created using the formula of the product of area and roundness, in which smaller values indicate a decrease in cell size relative to cell roundness^38^. We found that HeLa-Empty-GFP/LifeAct cells had larger values for measures of perimeter, area, roundness and CAF than Hela-CDH26A/LifeAct cells (Fig. 4c, d)-indicating expression of CDH26A in HeLa cells conferred cellular shape changes associated with cortical actin structures. Because CDH26A immunolocalizes near the plasma membrane in AECs, we explored whether this localization also occurred in HeLa cells overexpressing CDH26A. Using plasma membrane staining with CellMask Deep Red, and super-resolution microscopy to visualize single cells, we found co-localization of CDH26A and actin in the plasma membrane region of the HeLa-CDH26A/LifeAct cells (Fig. 4e). To determine the proximal relationship between CDH26A and actin in the cytoskeleton, we used Imaris 3D-rendering software to examine the relationship between fluorescent proteins fused to either full-length CDH26A (CDH26A-Clover-N1) or actin (mRuby2-LifeAct) in HeLa cells which were imaged by super-resolution confocal microscopy. We then determined whether co-localization of these two proteins occurred  off of these super-resolution images using  co-localization algorithms built into the Imaris software package. We found strong co-localization between CDH26A and actin in these experiments (Fig. 5a, b), and robust statistical associations using the Pearson’s correlation coefficient^39^ and Mander’s overlap coefficient^40^ (Fig. 5c). We also used Förster Resonance Energy Transfer (FRET) fluorescent microscopy to confirm the close proximity between the fluorescently labeled CDH26 A and actin (Fig. 5d, e).

### CDH26A maintains epithelial cell–cell contact

**Fig. 6:**
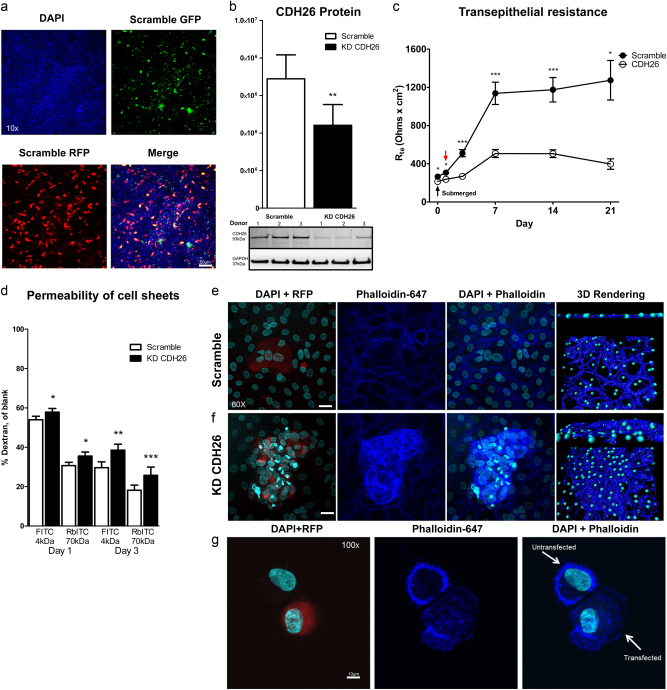
CDH26 maintains epithelial cell–cell contact through cortical actin structure. **a** Transfection efficiency of shRNA constructs in AECs after electroporation by monitoring overexpression of RFP or GFP. **b** Western blot data for CDH26A protein after knockdown of CDH26A by shRNA. *n* = 10 donors grown at ALI for 5 days and data presented as mean and s.e.m., statistics non-parametric paired *t*-test with Wilcoxon matched-pairs signed rank test where **p < 0.005 is significant when compared to scramble control. Bottom panel: western blot for anti-CDH26 from three representative donors after KD of CDH26A. GAPDH used as a loading control. **c** Transepithelial resistance (TEER) measurements from AEC with shRNA scramble control vs. KD CDH26A at different points in culture where each data point represents *n* = 3 donors with 6 replicates from each donor and data presented as mean and s.e.m., statistics non-parametric paired *t*-test at each time point with Wilcoxon matched-pairs signed rank test where *p < 0.05 and ***p < 0.0001 is significant when compared to scramble controls. Submerged corresponds to 24 h after plating cells before taking cells to ALI and the red arrow represents measurements of TEER 24 h after the initiation of ALI. **d** Cellular permeability assay with dextran tracers in submerged cells or cells after 2 days at ALI where each data point represents *n* = 3 donors with 6 replicates from each donor and data presented as mean and s.e.m., statistics non-parametric paired *t*-test, with Wilcoxon matched-pairs signed rank test where *p < 0.05, **p < 0.005 and ***p < 0.0001 significant to scramble controls. Pattern of actin staining visualized by phalloidin-647 in scramble control sheets (**e**) and KD CDH26A (**f**) grown at ALI for 5 days after transfection. **g** Actin phenotype of single-cell suspensions in untransfected cells and KD CDH26A cells in the same donors. Phalloidin-647 immunofluorescence is representative from survey of n = 3 donors and four fields per condition. Scale bar = 20 µm, unless otherwise noted.

To explore how loss of CDH26A affects the function of AECs, we knocked down CDH26A in primary human AECs using a plasmid library of five 29mer short-hairpin RNAs (shRNA) specific to variant A (Supplementary Figure S4). The shRNA plasmids also contained a separate CMV-driven promoter to over-express fluorescent protein in transfected cells, allowing us to track cells with the shRNA by their co-expression of either GFP or RFP. Using electroporation to introduce the shRNA plasmids into AECs, we found a transfection efficiency of 50–70% across donors (Fig. 6a). We achieved continual CDH26A suppression through expression of the shRNA constructs by a mammalian-driven promoter U6^41^. In imaging studies of the AEC sheets, we observed isolated regions of shRNA-transfected cells surrounded by non-transfected cells. Overall, we measured a ~12-fold decrease in *CDH26A* gene expression and a ~65% decrease in protein (Fig. 6b). In functional studies, we found marked decreases in transepithelial resistance (TEER) in shRNA-transfected cells (Fig. 6c). Specifically, the increase in TEER in the shRNA-transfected cells only reached 1/3 of the TEER in the scramble control cells throughout the measurement period. Paracellular permeability was also abnormal in shRNA-transfected cells, as evidenced by increased paracellular flux of dextran particles (Fig. 6d). Loss of cell junction integrity demonstrates that expression of CDH26A is necessary for proper monolayer formation and cell–cell contact. We explored whether cell death in transfected cells could account for the reduced TEER and permeability phenotypes in the CDH26A knockdown cells. Although Annexin V-647 was increased after CDH26A in KD cells, we consider this a result of membrane flipping related to the disruption of the overall organization of the cell sheets and not due to increased cell death (Supplementary Figures S5A and B). We did not see a significant decrease in the overall cell numbers between scramble and KD CDH26A nor was there a difference in cleaved caspase 3 staining among shRNA transfected cells and controls (Supplementary Figures S5C and D).

To determine the effect of CDH26A knockdown on actin organization in AECs, we stained the cells with phalloidin-647, a fluorescently labeled phallotoxin-derivative which binds to actin at a 1:1 ratio allowing visualization of the actin cytoskeleton by fluorescent microscopy. Using actin staining we found a marked loss of cortical actin structures and a highly disorganized cell sheet with cell clumping and absence of apicobasal organization (Fig. 6f). In isolated single epithelial cells, CDH26A knockdown was associated with markedly abnormal actin organization (Fig. 6g). Because increased cell migration is associated with tumor formation and we found the loss of actin organization upon knockdown, we explored if CDH26A has tumor suppressor properties. We found no significant increase in colony formation after loss of CDH26A. Knockdown of CDH26A did cause a statistical significant difference in cell proliferation in soft agar assays; however this is only a 220-unit difference in RFU and likely not biologically significant (Supplementary Figure S6).

### CDH26A expression is required for proper expression of planar cell polarity proteins

**Fig. 7:**
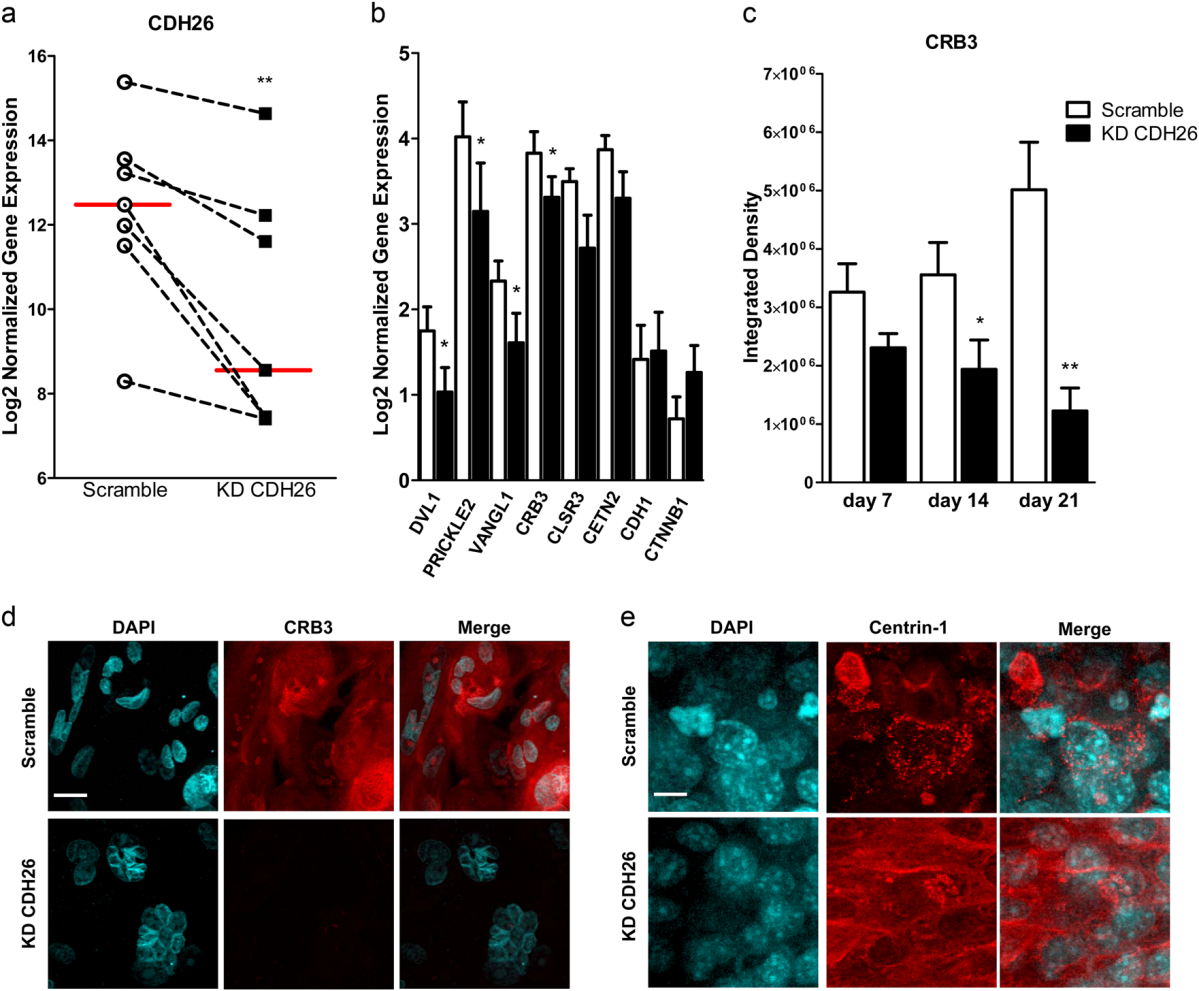
CDH26 expression is important for maintaining epithelial expression of planar cell polarity proteins. **a** CDH26 variant A gene expression by qPCR for samples at day 7 ALI confirming knockdown of CDH26A in *n* = 7 donors paired with scramble controls. Red bar-median expression of CDH26, dotted line represents the effects of knockdown within each individual donor. Statistics paired *t*-test where **p < 0.005 when compared to scramble controls. **b** Gene expression of proteins associated with planar cell polarity (PCP) by qPCR in the same *n* = 7 donors KD CDH26A, with data presented as mean and s.e.m. with statistics paired *t*-test where *p < 0.05 when compared to scramble controls. **c** Quantification of PCP protein CRB3 in AECs by immunofluorescence at different time points in ALI culture. **d** Expression and localization of CRB3 protein in scramble or KD CDH26A cells at 21 days ALI. **e** Expression and localization of centrin-1 protein in scramble or KD CDH26A cells at 21 days ALI. Images are representative from survey of *n* = 3 donors and 3 fields per condition, data presented as mean and s.e.m. with statistics non-parametric paired *t*-test with Wilcoxon matched-pairs signed rank test where *p < 0.05 and **p < 0.005 significant to scramble controls. Scale bar = 10 µm.

The localization of CDH26A to the apical region of AECs led us to consider if CDH26A has a role in planar cell polarity. To begin to explore this possibility, we examined whether CDH26A knockdown alters expression of planar cell polarity proteins such as DVL1 (dsh homolog 1), PRICKLE2 (Prickle Planar Cell Polarity Protein 2), VANGL1 (Van Gogh homolog), CRB3 (crumbs homolog 3), CLSR3 (flamingo1 homolog), CETN2 (Centrin-2), CDH1 (E-cadherin), and CTNNB1 (β-catenin). We selected this panel of genes based on the relationship of the actin cytoskeleton on apical expression and localization of planar cell polarity proteins in ciliated cells^42, 43^. For these experiments, we examined gene expression for PCP proteins at 7 days after CDH26A knockdown in AECs. After confirming the knockdown of CDH26A mRNA with qPCR (Fig. 7a), we found it was associated with significant decreases in DV1, PRICKLE2, VANG1, and CRB3 gene expression (Fig. 7b). CENT2 and CLSR3 expression was also decreased, but not significantly so. There was no change in CDH1 expression and non-significant increase in CTNNB1 after knocking down CDH26A. Knockdown of CDH26A did not significantly alter expression of three housekeeping genes (mean expression scramble vs. KD CDH26A: EEF1A1 0.78 ± 0.09 vs. 0.57 ± 0.14, PPIA 0.92 ± 0.23 vs. 0.60 ± 0.14 and RPL13A 2.59 ± 0.22 vs. 2.74 ± 0.07). As CRB3 is a key protein mediator in the transcriptional regulated Hippo pathway of planar cell polarity in the airway of mice^44^, we sought to determine the effect of loss of CDH26A on CRB3 protein. We found that CDH26A knockdown was associated with a marked decrease in CRB3 protein expression (Fig. 7c), where at 21 days at ALI the CRB3 localization was diffuse and apical in scramble controls and virtually absent in KD CDH26A cultures (Fig. 7d and Supplementary Figure S7). CDH26A knockdown was also associated with a loss of expression of DVL1, PRICKLE2 and VANGL1, which are PCP proteins associated with ciliogenesis^45^. This finding prompted us to examine the effect of CDH26A knockdown on the phenotype of cilia. Using centrin-1 as a cilia marker^46^, we found that CDH26A knockdown in AECs was associated with a marked decrease in cilia by the absence of punctacte ciliated structures at the apical membrane (Fig. 7e).

## Discussion

Although CDH26 is known to be expressed by AECs, its functional role in these cells is largely unknown. A recent study in gastrointestinal epithelial cells showed that CDH26 binds α4 and αE integrins to regulate leukocyte adhesion and activation^20^, but little is known about whether CDH26 has typical cadherin-like functions in AECs. We show here that a variant of CDH26 (CDH26A) is highly expressed in human AECs, localizes to the apical membrane, has functional cadherin domains and plays a previously unsuspected role in mechanisms of planar cell polarity in AECs.

In initial experiments we showed that a specific CDH26 variant (*CDH26* variant A) is highly expressed in primary human AECs but that robust CDH26A expression occurs only in primary cells cultured at ALI. These findings and the low or absent CDH26A expression in epithelial cell lines that cannot form well-differentiated ciliated cells in culture led us to focus our functional experiments on the role of CDH26A in establishment of epithelial cell polarity in the airway epithelial tissue plane. Key to establishing this polarity is asymmetric partitioning of planar cell polarity components in the apical vs. basal cell regions and the formation of cortical actin^47^.

Although E-cadherin is known to promote cell–cell contacts in AECs by cadherin-based formation of cortical actin^48^, we show here that CDH26A has separate and non-redundant roles in actin biology in epithelial cells. Whereas E-cadherin is important for stabilizing the actin at the zonula adherens^49^, we provide multiple lines of evidence to show that CDH26A plays a role in planar cell polarity by anchoring actin near the apical surface rather than at the cell junction. First, we show that CDH26A immunolocalizes to the apical region of AECs in both airway tissue and in cultured AECs. Second, in cell culture experiments where we knocked down of CDH26A in AECs, we show a loss of cortical actin and a loss of a typical epithelial cell phenotype. Formation of cortical actin is required for cells to form an organized epithelium as it creates a scaffold to orient structures to cell surface^48, 50^. Third, in experiments in which we overexpressed CDH26A in HeLa cells, we show an increase in formation of cortical actin that drives the cells to an epithelial cell-like phenotype. Together, these data provide strong evidence that CHD26A is a key regulator of the actin cytoskeleton and of the AEC phenotype.

A tightly regulated network of polarity proteins oriented in an apicobasolateral manner is required to facilitate cell and tissue organization^51^. Normal cell adhesion and polarity is highly dependent on the formation of cell structures necessary for cell–cell contact and recruitment of these complexes. Our proposal that CDH26A has a role in differentiation of AECs is novel, but it is supported by studies of miR-200b in mice. miR-200b regulates CDH26 expression and AECs from mir-200b−/− mice show decreased expression of CDH26 and a fibroblast-like phenotype^52^. In addition, it is not unlikely that CDH26A could play a key role in epithelial cell differentiation. Other atypical cadherin proteins, such as CDH23, are apical proteins with roles in planar cell polarity in tissue and isoform-specific ways^53, 54^.

Our data suggest that the mechanism by which CDH26A regulates epithelial cell differentiation is via its binding to actin. We showed data that we generated from pull-downs and western blots strongly suggest binding of CDH26A to actin. In addition, using super-resolution microscopy to visualize single cells, we found co-localization of CDH26A and actin in the apical plasma membrane. Imaris 3D-rendering and FRET co-localization studies confirmed the close proximity of CDH26A and actin. Taken together, these data suggest direct binding of CDH26A to actin rather than indirect binding via intermediary binding partners. Thus, we conclude that CDH26A binds actin to regulate the cytoskeleton of AECs.

We found evidence that that binding to the actin cytoskeleton of AECs influences planar cell polarity events in these cells. For example, the expression of multiple planar cell polarity genes in AECs was decreased upon CDH26A knockdown. Mislocalization of PCP complexes lead to cells with fibroblast-like phenotype due to loss of cell–cell contact and polarity^55^. Our findings mirror those that show that knockdown of planar cell polarity proteins such as Vang2 and Rac1 lead to loss of cortical actin structures^56^. Among these genes were several encoding PCP proteins that are associated with ciliogenesis^45^ and we found loss of these proteins were not associated with changes in E-cadherin or β-catenin expression. While we were unable to generate complete knockdown throughout the AEC cell sheets, the effects of CDH26A loss on polarity proteins such as CRB3 and centrin-1 also impacted neighboring non-transfected cells. In this regard, it is notable that we found that CDH26A knockdown in AECs is associated with a marked decrease in cilia.

Planar cell polarity is regulated at the transcriptional level in two independent manners. First, activation of transcription factors can directly activate PCP pathways. Wnt5 signaling can be directly activated by transcription factors, such c-Jun and ATF2^57^. In contrast, PCP can be activated by accumulation of proteins at the plasma membrane—such is the case with Wnt11 signaling where it interacts with protein Fz7 to cause Dvl (Dsh) accumulation^58^. Additional experiments will be needed to further explore how CHD26A influences planar cell polarity proteins in AECs. However, our studies provide strong rationale for further studies. We speculate that CDH26A is required for accumulation of PCP proteins at the apical surface.

In summary, we conclude that CDH26A promotes formation of cortical actin and directs apicobasal polarity in AECs. We propose that CDH26A is required for the formation of the actin cytoskeleton and the correct orientation of cell structures to the apical surface. CDH26A is therefore a previously unrecognized regulator of planar cell polarity in AECs.

## Materials and methods

### Sources of airway epithelial cells

For RNA-Seq, human AEC were isolated from tracheas collected from 141 cadaveric lung donors from the California Donor Network and grown at ALI for at least three weeks in culture, as a part of a separate study^24^. For knockdown experiments, AECs were grown using standard ALI methods as described previously^59^. Briefly, cells were expanded in 5% FCS in DMEM/F12 supplemented with a rho kinase (ROCK) inhibitor to promote proliferation^60^. Once cells reached 90% confluency, flasks were washed and cells were trypsinized with TrypLE. CDH26A was silenced by transfecting shRNA plasmids using the Neon transfection system, as detailed in the Supplementary Information.

### Characterization of CDH26 transcript variants by RNA-seq

Transcript models (top of Fig. 1a) were plotted using GenomeGraphs R package v3.3^61^. Whole transcriptome RNA-Seq libraries were generated with a modified KAPA protocol and sequenced as single end reads on Ion Torrent Proton. Isoform expression was quantified using kallisto (v0.42.3)^62^, with iGenomes hg38 reference transcriptome (downloaded 07/19/2016 http://support.illumina.com/sequencing/sequencing_software/igenome.html). We measured expression of the two canonical CDH26 isoforms (NM_177980 and NM_021810) in units of transcripts per million (TPM), which gives the number of times a particular transcript, normalized by transcript length, would appear in a sample of 1 million transcripts. This measure allows direct comparison of the relative abundances of transcripts in a sample. In addition to calculating TPM across the 141 samples, we also measured total library size per sample by summing the raw gene counts from across all transcripts.

### Characterization of CDH26 transcript variants by DNA gel

RNA was isolated from trachea donors or from cell lines using RNeasy Mini Kits (Qiagen) and RNA was quantified by nanodrop. To amplify transcript variant CDH26A by end point PCR, cDNA was generated from 500 ng of universal lung RNA (Agilent), fresh trachea tissue, AECs grown on transwells, or clonal cell lines using SuperScript Vilo cDNA synthesis kit (ThermoFisher). Touchdown qPCR for transcript variants was performed using hotstart using Advantage 2 RT-PCR kit (Clontech) for 40 cycles using methods detailed previously^63^. Expression of transcript variants was visualized using a 1.5% TBE agarose gel with UV transillumination on ImageQuant LAS4000. Primers and probes and a diagram detailing time points collected in culture are in Supplementary Figure S1.

### Western blots

AEC or clonal cell line lysates (10 μg) were denatured in 1% NP lysis buffer and run under reducing conditions on a 4–10% Bis-Tris gel in MES buffer (ThermoFisher). Membranes were blotted with 1:1000 anti-CDH26 (ProteinTech 20057-1-AP) or anti-StrepII 1:3000 (Gene Script 95040-994994) overnight. Anti-GAPDH (Ambion AM4300) 1:4000 was used as a lysate loading control. Membranes were incubated with secondary HRP antibody (Vector Laboratories) at 1:10,000 and imaged using chemiluminescence on ImageQuant LAS4000. Analysis of band intensity between conditions was performed using ImageJ measurement tool of integrated density as a non-biased measurement of signal intensity, as previously described^64^.

### Immunofluorescence

Staining of trachea biopsies from cadaveric donors or AECs cells grown on transwell inserts was performed with standard methods; antibodies and protocol details are provided in the Supplementary Information.

### Overexpression of CDH26A in clonal cell lines

Cells were transfected using Neon electroporation system (ThermoFisher). Where stable cell lines were generated, cells were selected with G418/neomycin. CHO-K1 cells were grown in F12-K media supplemented with 10% FCS. Cells were transfected and selected to stably express turboGFP-tagged CDH26A for aggregation assays. For migration and adhesion assays, HeLa cells were transiently transfected with turboGFP-tagged CDH26A and grown in DMEM supplemented with 10% FCS. For measurements of transepithelial resistance, HeLa cells expressed StrepII-tagged CDH26A. HeLa cells were plated at a density of 200,000 cells/well on transwell 3460 and measurements were performed using a chopstick voltmeter. For assessment of cytoskeletal rearrangements HeLa cells were transiently co-transfected with turboGFP-tagged CDH26A or turboGFP-empty and mCherry-LifeAct to visualize actin^36^, nuclei stained for DAPI and visualized by confocal microscopy. For FRET measurements, clover-10-mRuby was used as a FRET control. HeLa cells were transiently co-transfected with clover-N1 or CDH26A-clover-N1 and mRuby-LifeAct-7. Plasmid maps are shown in Supplementary Figure S8 and vector sequences are provided in the Supplementary Information.

### Morphometric measurements on HeLa cells

To see cell shape change after transiently transfecting CDH26A into HeLa cells, fluorescent images with either Empty-turboGFP (Empty-GFP) or turboGFP fused to the c-terminus of CDH26A (CDH26-GFP) with mCherry-LifeAct were obtained at 60×. Nuclei were counterstained with DAPI. Single plane images were created from z-stacks using maximum intensity z-projections in ImageJ. Stably transfected HeLa cells were imaged in phase at 60×. Cell borders were outlined using the ImageJ macros Measure Cell Surfaces tool. Measurements were generated by measuring area, perimeter, roundness and circularity on individual cells using the ImageJ measurements tool.

### Measurement of co-localization in HeLa cells

Images were 3D rendered using Imaris 8.1. Co-localization was determined by setting a region of interest (ROI) field over cells expression either Clover-N1 or CDH26-Clover-N1 and mRuby2-LifeAct. Thresholding for channel 1 (Clover) and channel 2 (mRuby2) was set to be identical between fields. A rendering of the co-localized regions was generated (yellow panel) and measurements of correlation coefficients obtained for this region.

### Measurement of FRET in HeLa cells

The Förster Resonance Energy Transfer (FRET) was measured between FRET pair Clover (505 λex 515 λem) and mRuby2 (559 λex 600 λem). FRET parameters were using the FRET control, Clover-mRuby2-FRET-10 in HeLa cells. To measure FRET between CDH26A and actin, HeLa cells were transiently co-transfected with Clover-N1 or Clover fused to the c-terminus of CDH26A (CDH26-Clover-N1) with mRuby2-LifeAct. The fusion to the c-terminus of CDH26A resulted in fusion to the cytoplasmic region of CDH26A in closest proximity to the actin cytoskeleton. The donor Clover was imaged (pre-bleach) followed by acceptor mRuby2 photobleaching for 30 s. The donor Clover was reimaged (post-bleach) to measure the presence of increased fluorescent intensity in the donor after acceptor photobleaching. The percent FRET efficiency for the Clover-mRuby2 pair was measured on individual cells by using the integrated density measurement in ImageJ and the formula *I*_DonorPostBleaching_−*I*_DonorPreBleaching_/*I*_DonorPostBleaching_ × 100%^65^. In images, donor Clover intensity is presented using a 16-color LUT curve.

### CHO-K1 aggregation assays, HeLa adhesion and migration assays

Aggregation assays were performed in CHO-K1 cells as previously described^66^. Matrix adhesion and migration were performed with HeLa cells by standard methods described previously^67, 68^, and detailed in the Supplementary Information.

### Recombinant CDH26A protein synthesis and sedimentation assay

Recombinant CDH26A (rCDH26A) protein was purified by the University of North Carolina Protein Expression and Purification Core Facility. To determine whether rCDH26A formed multimers or macromolecular structures through self-binding, we modified sedimentation assays detailed previously^69^. The details of the protein purification methods and the sedimentation assays are provided in the Supplementary Information.

### CDH26 and actin pull-down

To pull-down CDH26A and potential binding partners, 50 μg of cell lysates from AECs grown at ALI for at least 3 weeks in culture, were incubated with 5 μg CDH26A antibody cocktail (Abnova PAB17494, Sigma SAB1306903 and HPA015722, Santa Cruz sc-85468 and sc-85446), actin (Santa Cruz sc-7210) or rabbit sera control and immunoprecipitated using Protein G-Agarose (Roche). Samples were split and half run for total protein using Flamingo Total Protein Stain (Biorad) or blotted for potential binding partners: p120/δ-1-catenin (Santa Cruz sc-13957), β-catenin (Santa Cruz sc-7963), α-e-catenin (Santa Cruz sc-9988).

### Knockdown of CDH26A in AECs

To silence CDH26A, four unique 29mer shRNA constructs in a retroviral RFP vector and one custom designed 29mer shRNA construct in a retroviral GFP vector were synthesized by Origene, vector and 29mer sequences are available Supplementary Figure S4. Detailed transfection methods to silence CDH26A are provided in the Supplementary Information.

### Parcellular flux assay

Tight junction integrity and paracellular permeability was measured using size-selective fluorescently labeled dextran, as previously described^70^. Human AECs transfected with shRNA to silence CDH26A or scramble control were taken to ALI. After reading transepithelial resistance, media was replaced with 5% FCS 250 μL on the apical membrane and 1 mL in the basal chamber and placed back into the incubator to equilibrate. An empty filter with no cells was used as a control for flux and 25 μL of rhodamine B-isothiocynate-dextran (RbITC) 70,000 mw and 25 μL of fluorescein isothiocynate-dextran (FITC) 4000 mw were added to the apical chamber for a final concentration of 2 mg/mL for each tracer. After 4 h, 100 μL was collected from the basal chamber to measure transit of tracer from apical to basal chamber and read (FITC ex 485 nm/em 544 nm, RbITC ex 520 nm/em 590 nm) in a black 96 well microplate on a Biotek Synergy H1 Multi-mode Microplate Reader.

### qPCR for planar cell polarity proteins

cDNA was generated from AECs RNA (20 ng) using SuperScript Vilo cDNA synthesis kit (ThermoFisher). Genes were preamplified for 15 cycles using hotstart using Advantage 2 RT-PCR kit (Clonetech). Measurement of DV1, PRICKLE2, VANGL1, CRB3, CLSR3, CETN2, CDH1 and CTNNB1 or variant-specific primers to CDH26A by qPCR using TaqMan Universal PCR Mix (ThermoFisher) on ViiA7 were normalized to epithelial housekeeping genes EEF1A1, PPIA and RPL13A as previously described^17, 71^. Details of the primers are provided in Supplementary Figure S9.

### Statistics and figure generation

Statistics and figure generation were performed using statistical software GraphPad Prism 5. Data were presented as mean and s.e.m. Non-parametric assumptions were made when comparing human airway epithelial cells grown at ALI. Comparisons made at different time points in ALI culture were made using the Kruskal–Wallis test with Dunn’s Multiple Comparisons test. Comparisons between paired scramble verse knockdown cells were made using Wilcoxon matched-pairs signed rank test. For clonal cell lines, paired *t*-test were used. All tests were considered significant where *p*-values are represented as *p < 0.05, **p < 0.005 and ***p < 0.0001. Vector maps were generated with SnapGene Viewer.

## Electronic supplementary material

Supplementary Information
