# Supplementary material for: Cadherin-26 (CDH26) regulates airway epithelial cell cytoskeletal structure and polarity
Source: Cell Discov. 2018 Feb 13;4:7. doi: 10.1038/s41421-017-0006-x (PMC5809386; doi:10.1038/s41421-017-0006-x)
Supplement: Supplementary file 1 — Supplementary Information [file 41421_2017_6_MOESM1_ESM.pdf]

SUPPLEMENTARY INFORMATION

Supplemental Methods

Immunofluorescence

Depariffinized trachea biopsies from cadaveric donors or AECs cells grown on transwell inserts were unmasked using HEIR in citrate buffer pH 6.2. Cell sheets were permeablized using methanol. Cells were stained for anti-CDH26 1:65 (Sigma HPA015722), anti-e-cadherin 1:50 (BD Biosciences 610181), acetylated tubulin 1:200 (Sigma 611B-1), anti-beta-catenin 1:50 (Santa Cruz sc-376959), anti-p120/δ-1-catenin (Santa Cruz sc-390327), anti-α E-catenin (Santa Cruz sc-9988), cleaved caspase 3 (Asp 175) (Cell Signaling Technology 9661S) or anti-CRB3 1:50 (Abcam ab180835). Secondary antibodies (Molecular Probes) Alexa 488, Cy3 or Alexa 647 were used at a concentration of 1:100. Nuclei were stand with DAPI 1:100. Annexin V-647 and phalloidin-647 staining was performed per the manufacturer protocols (ThermoFisher). To generate single cell suspensions for cytospins, cells were release from cell sheets using accutase to maintain cell viability and surface structures. Cell Mask Deep Red staining was performed per manufacturer protocols (ThermoFisher). Confocal microscopy was performed on a Nikon C1si Spectral Confocal as part of the UCSF Biological Imaging Development Center (BIDC) or on a confocal laser-scanning microscope Olympus Fluoview 10i. FRET and super resolution imaging were performed on a Zeiss LSM 880 Laser Scanning Confocal with Airyscan at the UCSF Gladstone Institute Microscopy Core. Analysis was performed using ImageJ measurement tool of

integrated density as a non-biased measurement of fluorescent intensity, as previously described [1]. 3D rendering was performed by Imaris 7.2 imaging software.

CHO-K1 Aggregation Assays

In brief, Empty-GFP or CDH26-GFP expressing cells were washed in Ca^{+2}/Mg^{+2} free HEPES and resuspended in Ca^{+2}-free HANKS buffered with HEPES and 1% BSA at a cell density of 5.0 x 10^4/mL. Cells were made into a single cell suspension by passing through a 21g needle and plated in a 24 well plate at a density of 250,000 cells per well. To determine if calcium enhanced aggregation, 2mM/L $CaCl_2$ solution was added and compared to wells containing no calcium. Cells were incubated for 90 mins at 37 °C at 5 x g on an orbital shaker. Assay was stopped by adding 5% PFA and large aggregates were photographed using green fluorescence filter on the ImageQuant LAS4000. Aggregation presented as % change increase above empty-GFP control.

Recombinant CDH26 Protein Synthesis and Sedimentation Assay

The full-length native human CDH26 protein, herein indicated as rCDH26 (amino acid start MAMRS, end SGVPS) was generated with a C-terminal dual StrepII tag (GSSAWSHPQFEKGGGSGGGSGGSAWSHPQFEK) by cloning into the mammalian expression vector pAlphaH (Supplemental Text S2) under the control of a CAG promotor. The protein was expressed using the mammalian expression system using EXPI293 cells (ThermoFisher) according to the manufacturers supplied protocols via transient transfection and the cell pellet collected by centrifugation 48 hours post transfection. Cells were lysed by sonication and protein purified using manufacturer

recommended protocols with a 1 mL of Streptactin Sepharose High Performance StrepTrap HP column (IBA Lifesciences). The sample was passed over an analytical superdex S200 10/300mm gel filtration column (GE Healthcare). The fractions containing rCDH26 were pooled elutions from this capture step and protein concentration determine ABS 280nm using calculated extinction coefficient calculated from the amino acid sequence. Final protein preparations were further purified by analytical size exclusion columns to generate a pool corresponding to the full-length protein of ~92kDa (see Supplemental Figure S3).

For samples in which protein sedimentation was assessed in calcium-free solutions, 15 µg of rCDH26 was concentrated in 3K Amicon Ultra filter units and buffer exchanged into calcium-magnesium free dPBS. Samples were subjected to incubation at room temperature for 1 h in the presence or absence of calcium to facilitate homotypic binding. Samples were then spun at 5,000g (slow), 20,000 g (fast) or 100,000g (UC-ultracentrifuge) at 22 °C for 20 mins. Supernatant liquid was removed and pellet left in the tube. Laemmli sample buffer was added to each, in-gel staining was done with Flamingo Total Protein Stain (Biorad) and western blots probed for 1:3000 anti-StrepII-HRP to detect StrepII-tagged rCDH26 in each fraction.

HeLa Adhesion and Migration Assays

For adhesion assays, Empty-GFP or CDH26-GFP cells were serum starved overnight. In 24 well plates, rat tail collagen was gelled in 10X HBSS for 60 minutes at 37 °C. Plates were blocked with 2% BSA for 30 minutes at 37 °C. Cells were seed at 100,000

cells per well in serum-free media and allowed to attach to the matrix for 1 hour at 37 °C. Unattached cells were removed by washing the surface of plate 3 times with PBS. Cells were visualized with crystal violet staining and counted. Migration performed in transwell 3422 rat tail collagen coated plates. Plates were washed and blocked with 0.1% BSA for 1 hour at 37 °C. Cells were serum starved overnight, trypsinized and resuspended in DMEM with 0.1% BSA at a cell density of 1 x 10^6 cells/mL. Cells were plated at a density of 100,000/well in the upper chamber and allowed to migrate to serum media with 0.5% FCS in DMEM for 6 hours at 37 °C. The GFP-positive cells that migrated through the filter pores were quantified using green fluorescence filter on the ImageQuant LAS4000.

Vector Cloning and Expression Systems

Over-expression in cell lines was performed using the Neon electroporation system (ThermoFisher). CDH26 (Myc-DDK-tagged)-Human cadherin 26 (CDH26), transcript variant A vector was purchased from OriGene. The insert for CDH26 was cut out using AsiSI (SgfI) and MluI (New England Biolabs) and cloned into entry vector pCMV6-AC-mGFP vector to generate a c-terminal fused mGFP-CDH26 protein. For the StrepII-tagged CDH26, CDH26 was synthesized from sequence optimized data and was cloned into vector backbone pAlphaH (formally known as pHLSec2) [2], by Genewiz using cloning sites KpnI and EcoRI (New England Biolabs). Using the pLL3.7 backbone [3, 4], LifAct-mCherry was cloned in place of GFP using NheI and EcoRI (gift from Mallar Bhattacharya, UCSF). Vector maps and sequence information in S3.

Knockdown of CDH26 in Airway Epithelial Cells

To silence CDH26, Origene, hairpin sequences are detailed in S5, synthesized four unique 29mer shRNA constructs in a retroviral RFP vector and one custom designed 29mer shRNA construct in a retroviral GFP vector. A non-effective 29-mer scrambled shRNA cassette in pRFP-C-RS or pGFP-V-RS vector was used as a negative control. Cells were transfected using the Neon electroporation system (Invitrogen). Briefly, primary airway epithelial cells were expanded in flasks and lifted with TrypLE (Gibco) at 80-90% confluence. Cells were resuspended in Buffer R at a density of 3.0 x 10^6/mL. Cells were pulsed using 6 µg shRNA or scramble mix (see Supplemental Figure S4) using the Neon 100 µL tip at a single pulse of 1200 V width 40 ms. Cells were plated at a density of 600,000 cells/well on transwell 3460 submerged for 1-3 days in media supplemented with ROCK to allow cells to recover. After the submersion phase, cells were switched ALI and maintained in 50/550 DMEM/F12 supplemented with 2% Ultroser G [5]. Transepithelial resistance and voltage was measured using a chopstick voltmeter.

Soft Agar Assay for Colony Formation and Cell Proliferation

To determine if knockdown of CDH26 resulted in anchorage-independent proliferation or transformation, soft agar assays were performed as previously described in 96-well and 35 mm dish format as previously described [6]. Briefly, human AECs were transiently transfected with shRNA pool and plated at a density of 4000 cells/well in 96 well black plates with agar gel made in the base. Cells were overlaid with a feeder 0.5% agar layer. Cells were allowed to recover in plates for 96 hours and the number of viable

cells was quantified on a plate reader by a Resazurin reduction assay per the manufacturer’s instructions (ThermoFisher). To assay colony formation, cells were plated at a density of 500,000 cells per 35mm glass dish with a gel base and feeder overlay. Feeder layers were changed in dishes once a week. Cells were fixed, stained with crystal violet dishes imaged after three weeks to count colonies. Colony counting was performed using ImageJ analyze particles plugin.

References

1. Burgess, A., et al., *Loss of human Greatwall results in G2 arrest and multiple mitotic defects due to deregulation of the cyclin B-Cdc2/PP2A balance.* Proc Natl Acad Sci U S A., 2010. **107**(28): p. 12564-9. doi: 10.1073/pnas.0914191107. Epub 2010 Jun 10.
2. Aricescu, A.R., et al., *Eukaryotic expression: developments for structural proteomics.* Acta Crystallogr D Biol Crystallogr., 2006. **62**(Pt 10): p. 1114-24. Epub 2006 Sep 19.
3. Rubinson, D.A., et al., *A lentivirus-based system to functionally silence genes in primary mammalian cells, stem cells and transgenic mice by RNA interference.* Nat Genet, 2003. **33**(3): p. 401-6.
4. Riedl, J., et al., *Lifeact: a versatile marker to visualize F-actin.* Nature methods, 2008. **5**(7): p. 605.
5. Sachs, L.A., W.E. Finkbeiner, and J.H. Widdicombe, *Effects of media on differentiation of cultured human tracheal epithelium.* In Vitro Cell Dev Biol Anim., 2003. **39**(1-2): p. 56-62.
6. Ke, N., et al., *One-week 96-well soft agar growth assay for cancer target validation.* Biotechniques., 2004. **36**(5): p. 826-8, 830, 832-3.

Supplemental Figures

Figure S1: CDH26 primers

Figure S2: CDH26 does not localize with other junctional proteins

Figure S3: Synthesis of recombinant CDH26

Figure S4: CDH26 shRNA Vectors

Figure S5: Annexin V-647 and Cleaved Caspase-3 Quantification After Transfection of AECs

Figure S6: CDH26 is not a tumor suppressor: KD of CDH26 does not promote colony formation or excessive over-proliferation in soft agar assays

Figure S7: Confirmation of Knockdown in AECs stained for CRB3 and Centrin-1

Figure S8: Over-expression and Fluorescent Vectors with Sequences

Fig S9: qPCR Primers for Housekeeping Genes and Planar Cell Polarity Proteins

A.

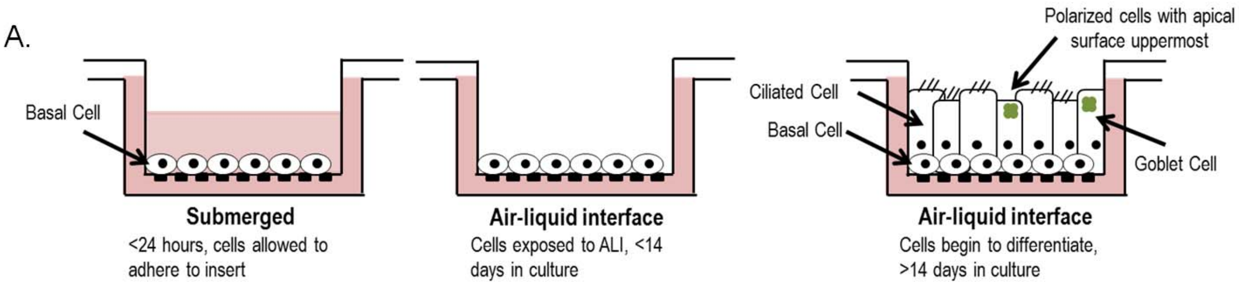

B.

Description	Name	Oligo Sequence
Forward 1	CDH26 NM_021810 TranscriptB FP	GATGACTAAGACGCAATTTGGCC
Reverse 1	CDH26 NM_021810 TranscriptB RP	TCCCTGAATAGTGATTCCCTTCC
Reverse 2	CDH26 NM_177980 Ex 1-8 RP	CCTTCGTTGGTCTCAGGGTCAG
Forward 2	CDH26 NM_177980 Ex1-8 FP	AGCTTACAAGTCAGCCCACCCC
Forward 3	CDH26 NM_177980 Ex9-18 FP	CAAAGCCTCATCATTGTCGTGG
Reverse 3	CDH26 NM_177980 Ex9-18 RP	TTCCAAGCTGGCCAGAGAGC

C.

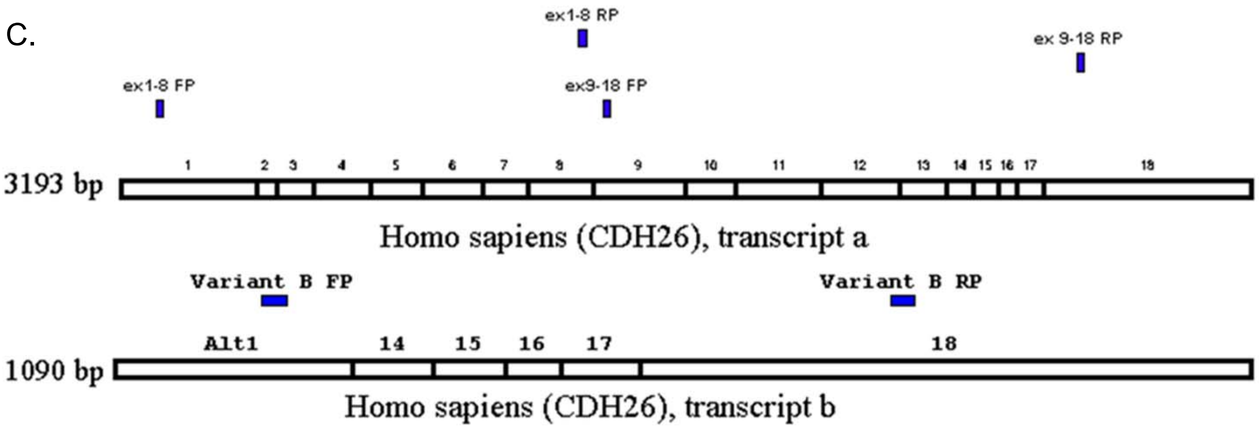

D.

Description	Name	Oligo Sequence
Forward RT	CDH26 NM_177980 Forward	TGGAGACAAACAGGGACTTTCC
Reverse RT	CDH26 NM_177980 Reverse	AAGCCCCACTTCTGCATCTG
TaqMan Fwd	CDH26 NM_177980 TM Forward	TCCCAGAAGCAAACTGTCCAT
TaqMan Rvs	CDH26 NM_177980 TM Reverse	CATCTGCAAGCTCCACACATG
TaqMan Probe	CDH26 NM_177980 TM Probe	CCACTGGCACAGGGGCAGATCCTT-FAM(BHQ)

E.

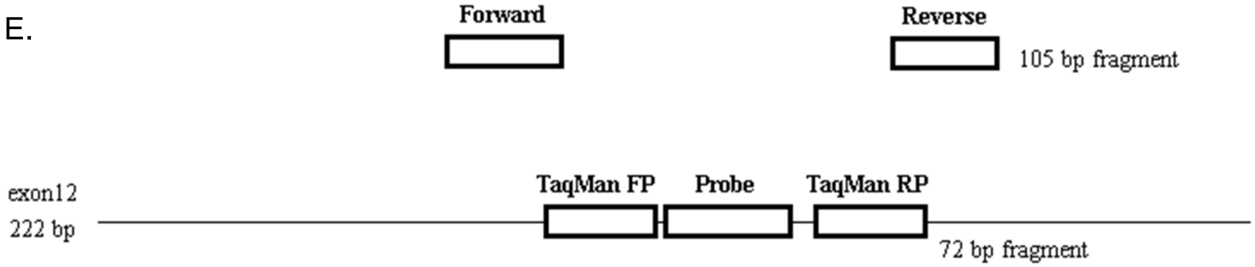

Figure S1: CDH26 primers. (A) Schematic of time points in culture AECs were harvested for CDH26A transcript measurements. (B) Primer sequences for amplification of variant specific exonic regions of CDH26 transcript variant A and B. (C) Map of amplification regions for variant detection of mRNA transcript by DNA gel. (D) Primer sequences generated for CDH26_A specific qPCR assay. (E) Map of amplification regions for CDH26_A qPCR residing in exon 2.

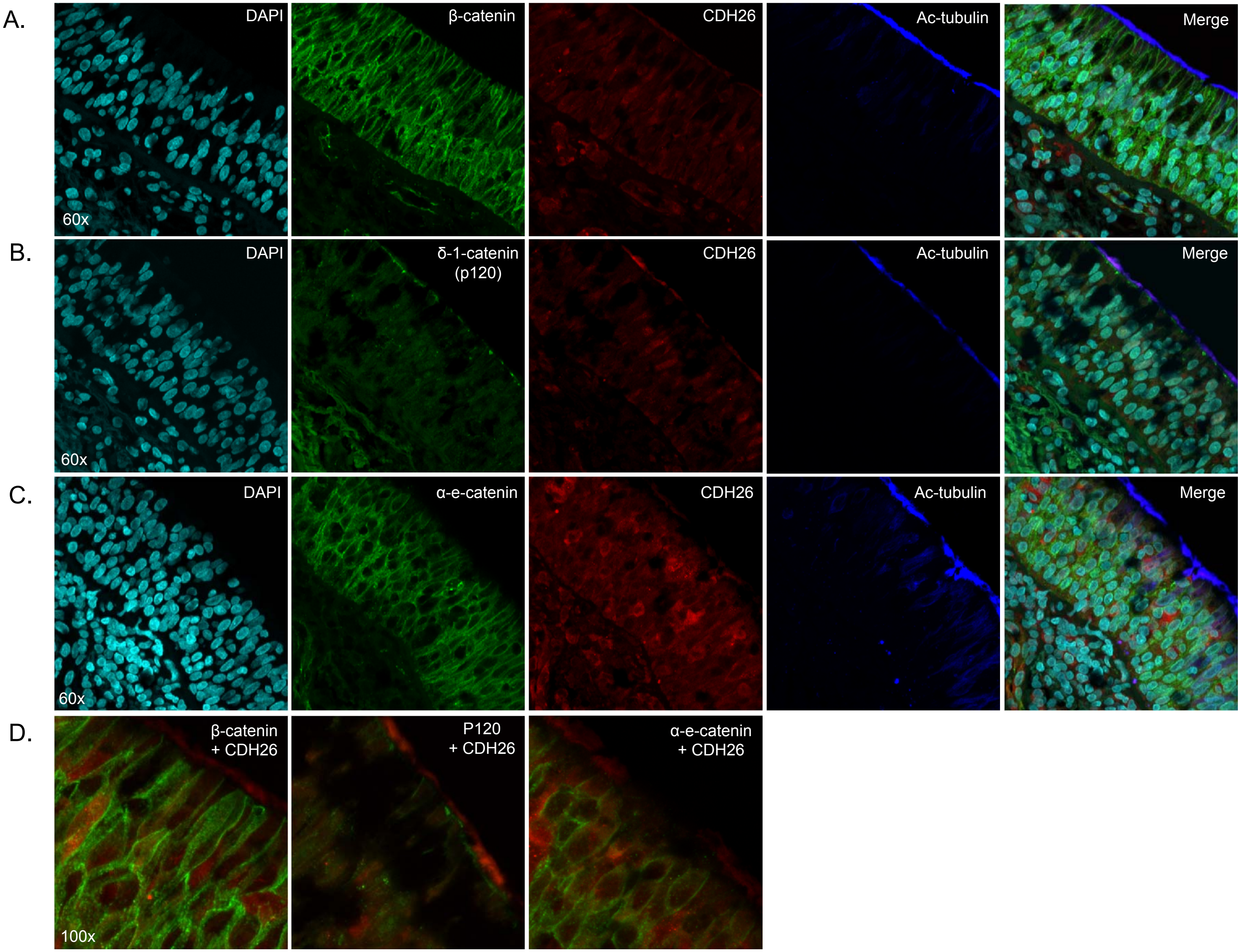

Figure S2: CDH26 does not localize with other junctional proteins. Immunofluorescence for junctional proteins (A) β-catenin (B) δ-1-catenin (p120) and (C) α-e-catenin in biopsies from human tracheas. (D) Higher magnification regions showing that CDH26 is non-junctional and predominantly in the apical membrane of cells. ..

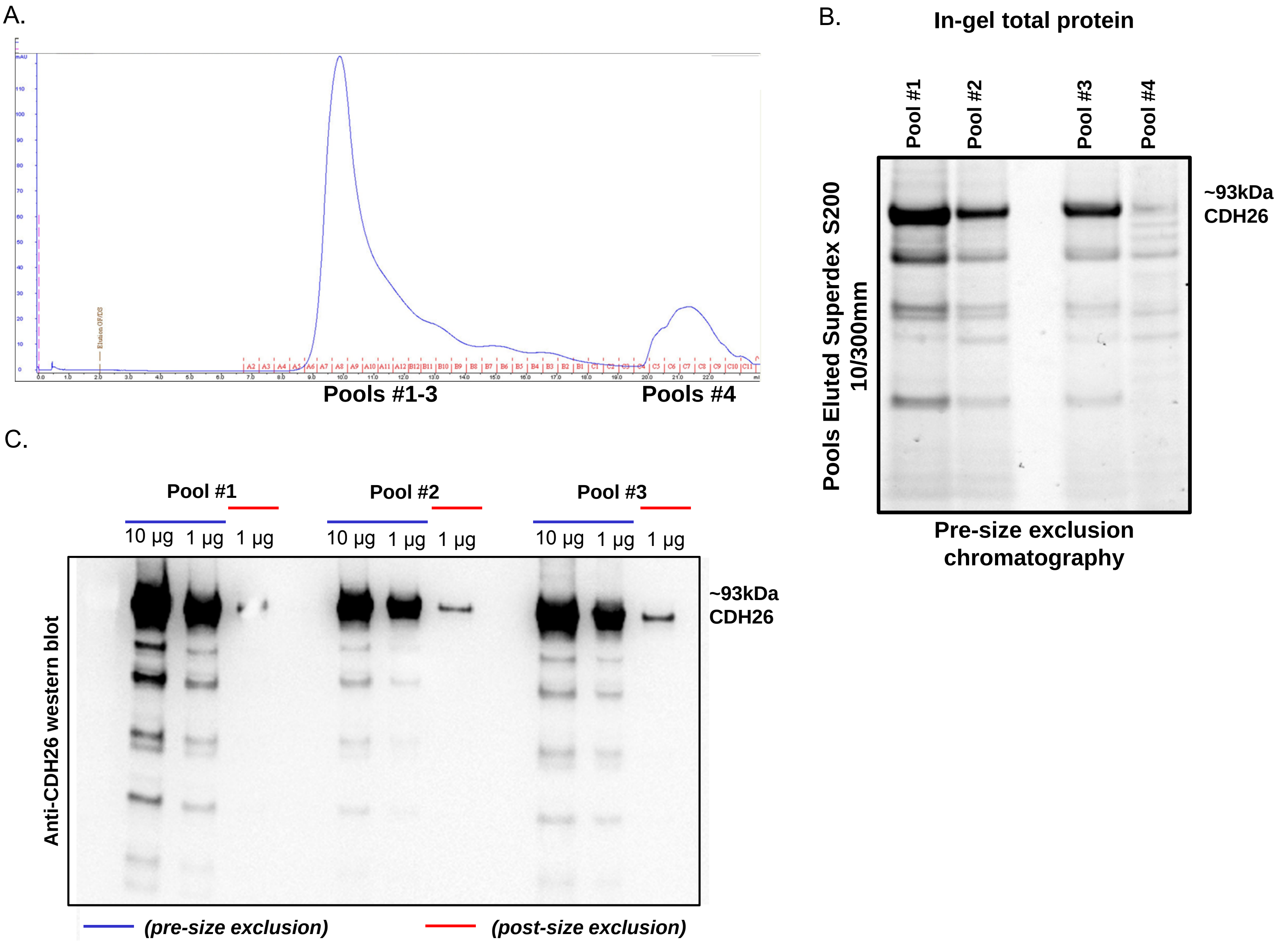

Figure S3: Synthesis of recombinant CDH26 (A). The fraction eluted from Expi293 lysates, corresponding to pools 1-3, are near the void of the column indicating multimeric products 220-660 kDa. Pool 4 represents small molecular weight products the end of the column with little CDH26A. (B). In-gel total protein stains show pools eluted from purification column void fraction correspond to mainly CDH26, with some smaller molecular weight contaminants, likely degradation products. (C) Western blot for anti-CDH26 on rCDH26A show size exclusion chromatography cleaned up products yielding purified protein..

A.

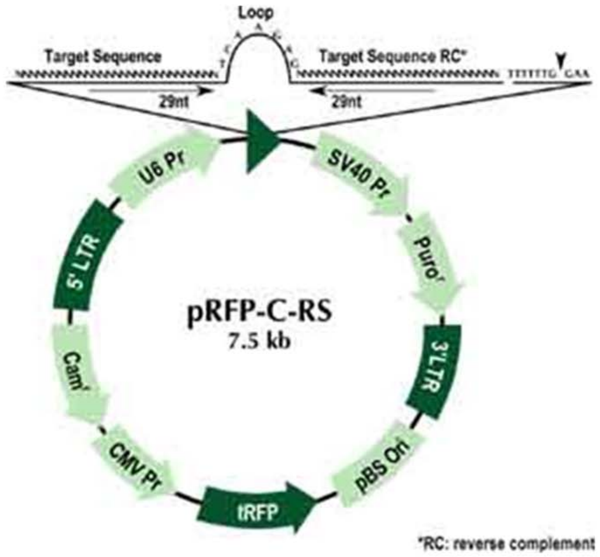

B.

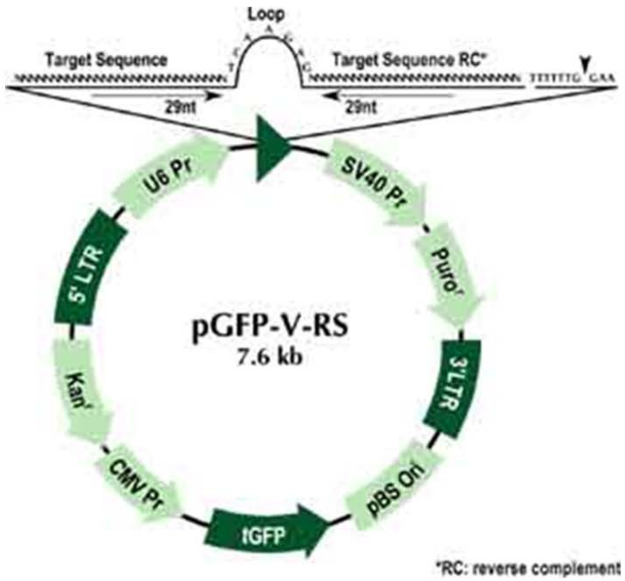

shRNA sequences
FI321905 CCTAATGCTCTTCCTGTCTGACATCAATG
FI321906 CTCTGGCTGCTTAGATTATGAGACCGCTC
FI321907 ATAATGCGGAGAGCAAAGGCACTTCAGCC
FI321908 TGGTGCCACTCTTCATTGGAGACAAACAG

shRNA sequences
HT125421A TAAGGTTCAGATTCCTGAAGGCCGAGCCA

C.

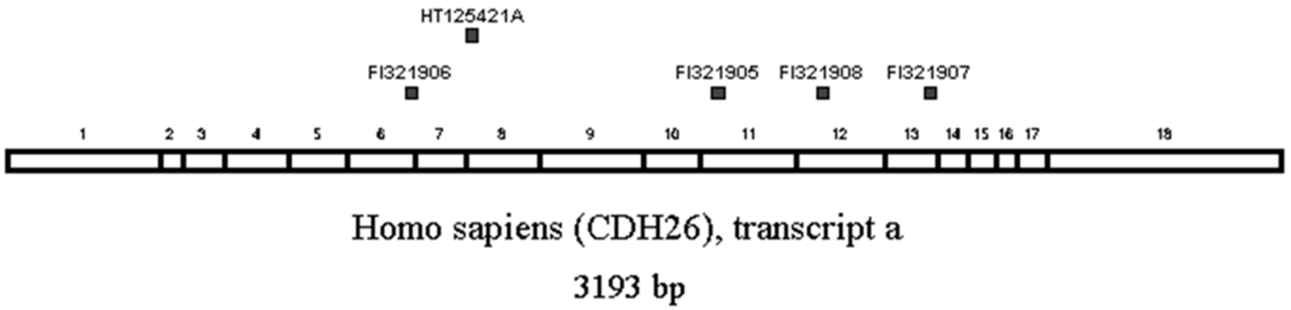

D.

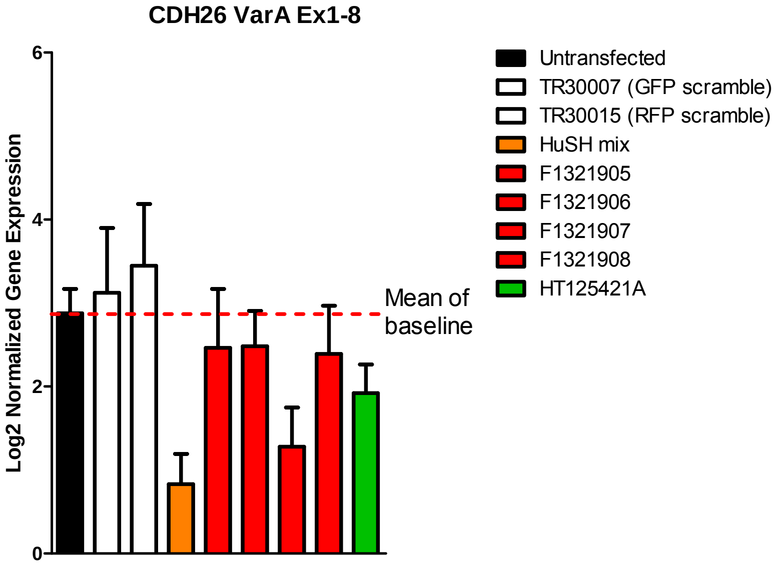

Figure S4: CDH26A shRNA Vectors. Five shRNA sequences were designed (A & B), against regions in exon 6, exon 7, exon 11, exon 12 and exon 13—regions within CDH26 variant A for variant-specific gene silencing. (C) Exon map where shRNA sequences fall on the CDH26A transcript. (D) qPCR for expression of CDH26 after individual hairpin qPCR in n=7 AECs donors showing the efficiency of each individual hairpin verses pooling all hairpins and no loss of CDH26A expression using the scramble control hairpin vectors.

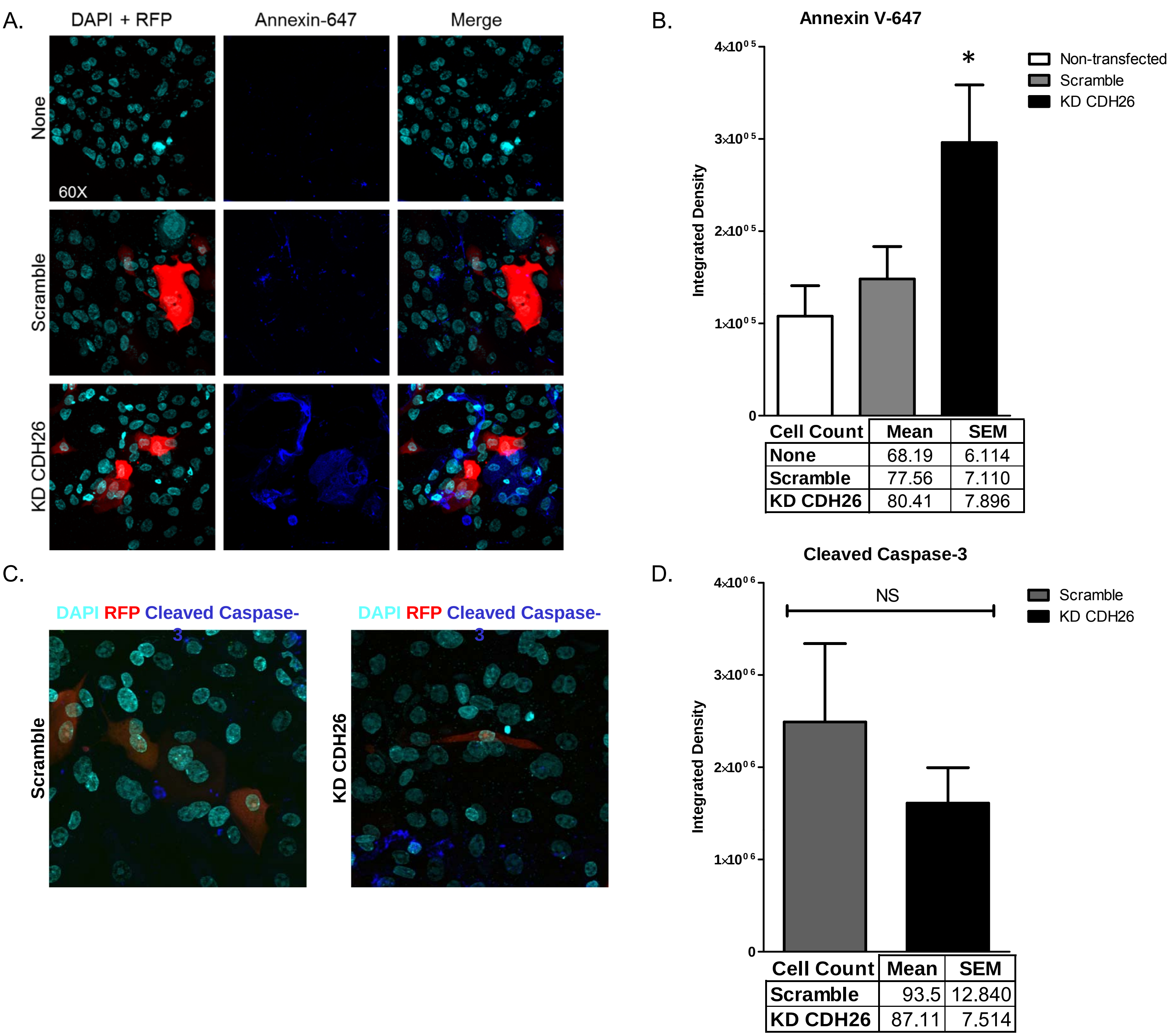

Cell Count	Mean	SEM
None	68.19	6.114
Scramble	77.56	7.110
KD CDH26	80.41	7.896

Cell Count	Mean	SEM
Scramble	93.5	12.840
KD CDH26	87.11	7.514

Figure S5: Annexin-647 and Cleaved Caspase-3 Quantification After Transfection of AECs. (A) Increased Annexin-v staining was observed after KD of CDH26 in AECs. (B) Increased Annexin-v staining was not associated with loss of cells, cell counts were not different between untransfected, scramble controls and KD CDH26 cells.(C) Cleaved caspase-3 staining was not significant (D) between scramble control's and KD CDH26 cells.

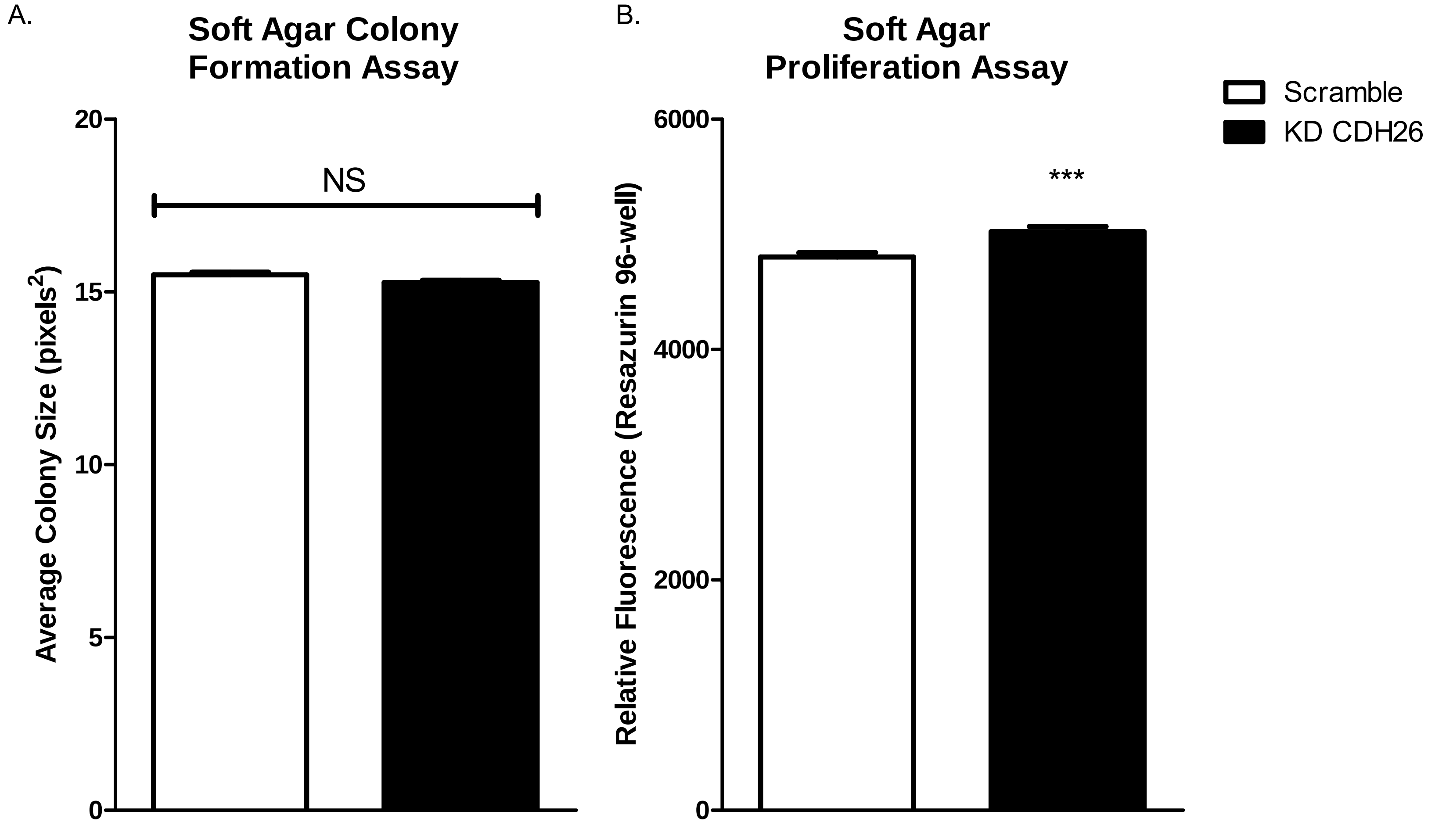

Figure S6: CDH26 is not a tumor suppressor: KD of CDH26 does not promote colony formation or excessive over-proliferation in soft agar assays. (A) KD of CDH26A does not promote colony formation in AECs. (B) KD cause a statistical significant difference in over-proliferation in soft agar assays**;** however this is only a 220-unit difference in RFU and likely not biologically significant.

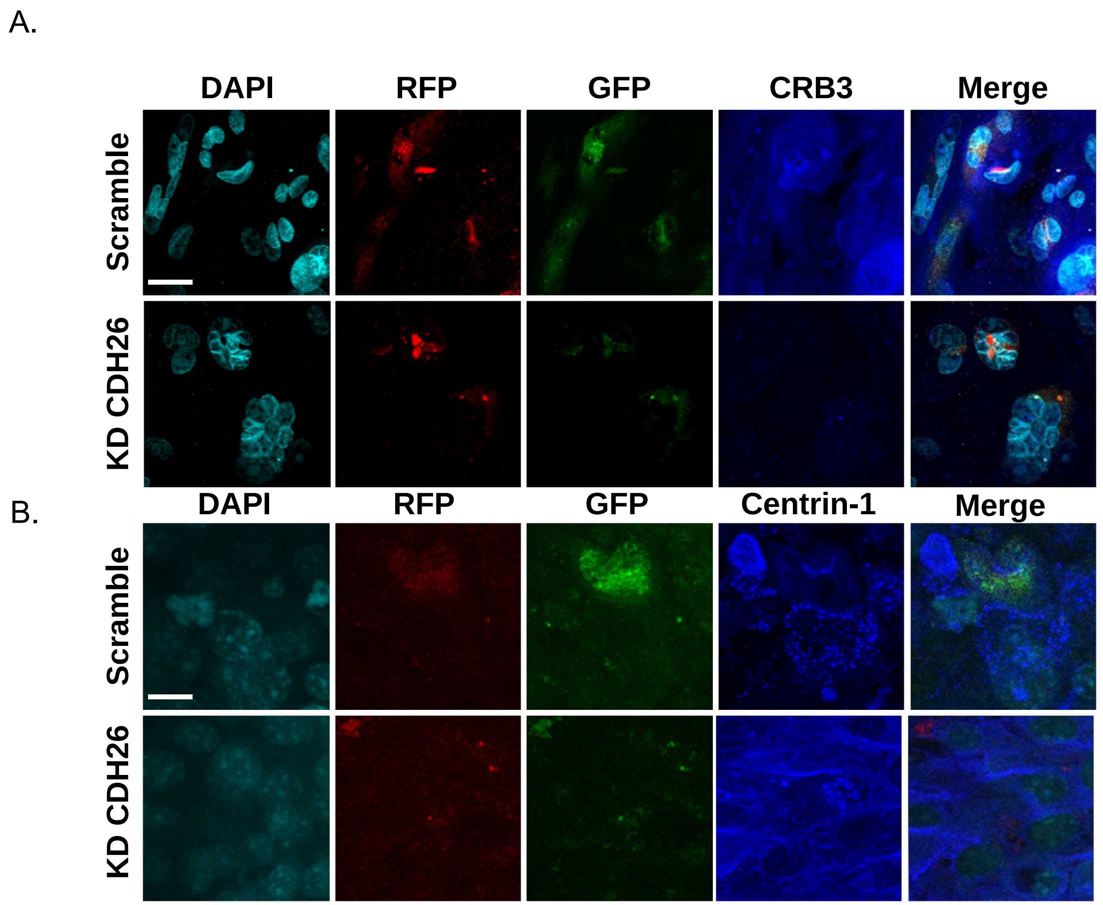

Figure S7: Confirmation of Knockdown in AECs stained for CRB3 and Centrin-1 (A) Expression and localization of CRB3 protein in scramble or KD CDH26A cells at 21 days ALI showing uniform CRB3 staining in scramble controls and the absence of CRB3 staining in KD CDH26A cells. (B) Expression and localization of centrin-1 protein in scramble or KD CDH26A cells at 21 days ALI showing punctate apical expression of centrin-1 corresponding to ciliary region and diffuse centrin-1 staining in KD CDH26A cells. Images are representative from survey of n=3 donors and 3 fields per condition. Scale bar = 10 µm.

Figure S8: Expression and Fluorescent Vectors.

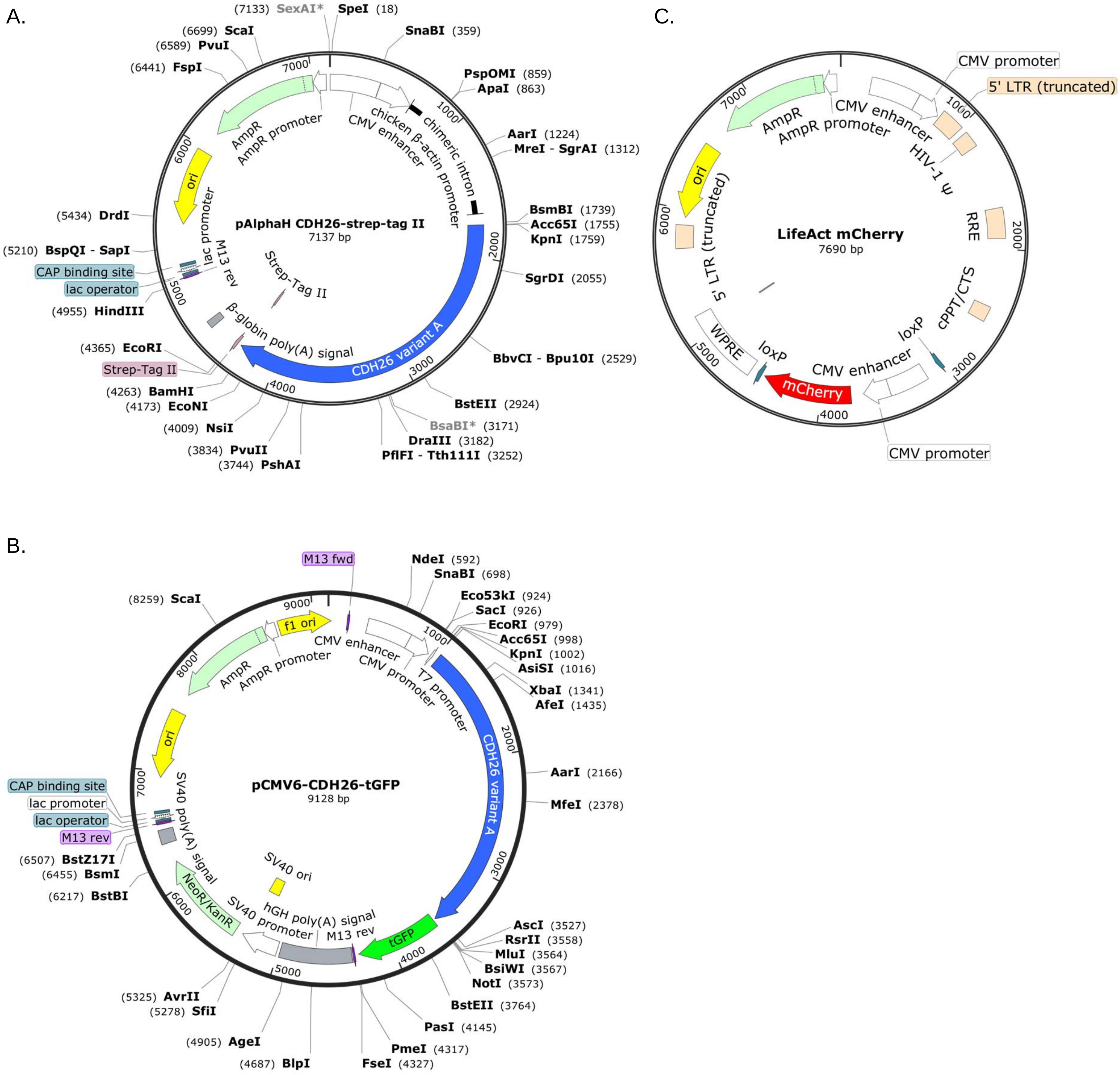

D.

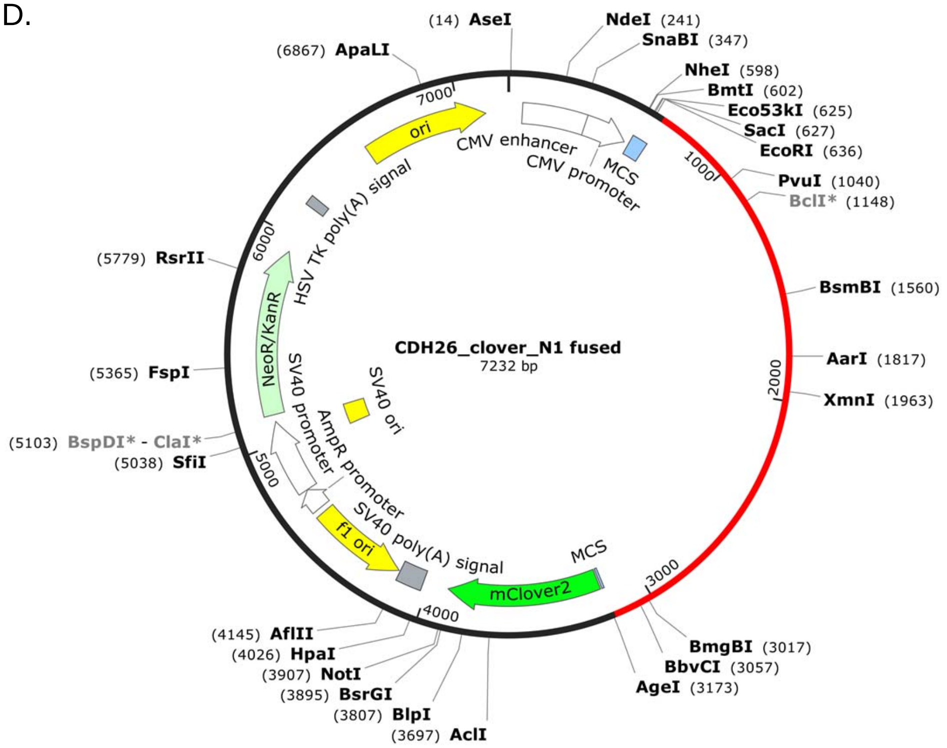

E.

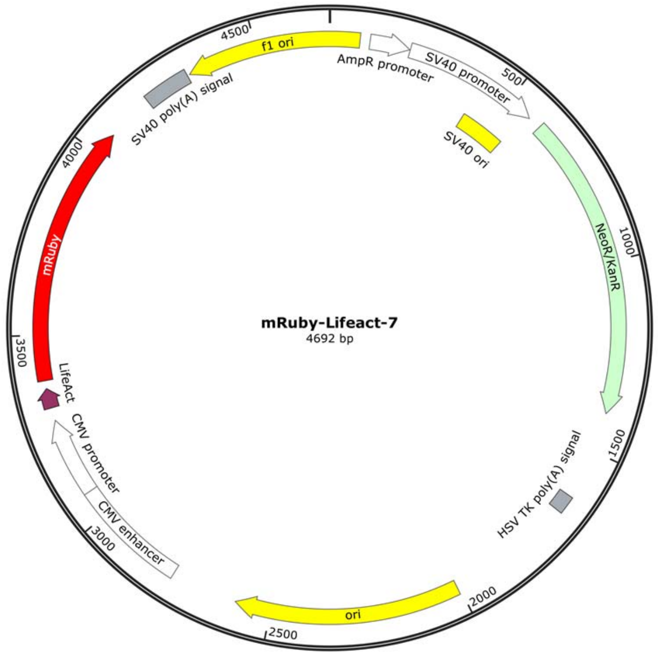

Figure S8: Expression and Fluorescent Vectors (A) StrepII-tag CDH26A overexpression vector. (B) C-terminal turboGFP-CDH26A fusion expression vector. (C) mCherry-tag LifeAct peptide expression vector actin visualization. (D) FRET vector generated with a C-terminal clover fusion to CDH26A. (E) FRET mRuby pair with LifeAct for actin visualization.

Supplement S8 Overexpression Vector Sequences

> PalphaH_CDH26-StrepII 7137 bp KpnI highlighted in Yellow 5'UTR highlighted in Green EcoRI highlighted in Pink Inserted Sequence in Blue

GTCGACATTGATTATTGACTAGTTATTAATAGTAATCAATTACGGGGTCATTAGTTCATAGCCCATATATGGAGTTCCG
CGTTACATAACTTACGGTAAATGGCCCGCCTGGCTGACCGCCCAACGACCCCCGCCCATTGACGTCAATAATGACGT
ATGTTCCCATAGTAACGCCAATAGGGACTTTCCATTGACGTCAATGGGTGGACTATTTACGGTAAACTGCCCACTTGG
CAGTACATCAAGTGTATCATATGCCAAGTACGCCCCCTATTGACGTCAATGACGGTAAATGGCCCGCCTGGCATTAT
GCCCAGTACATGACCTTATGGGACTTTCCTACTTGGCAGTACATCTACGTATTAGTCATCGCTATTACCATGGGTCGA
GGTGAGCCCCACGTTCTGCTTCACTCTCCCCATCTCCCCCCCCTCCCCACCCCCAATTTTGTATTTATTTATTTTTTAA
TTATTTTGTGCAGCGATGGGGGCGGGGGGGGGGGGGGCGCGCGCCAGGCGGGGCGGGGCGGGGCGAGGGGCG
GGGCGGGGCGAGGCGGAGAGGTGCGGCGGCAGCCAATCAGAGCGGCGCGCTCCGAAAGTTTCCTTTTATGGCGA
GGCGGCGGCGGCGGCGGCCCTATAAAAAGCGAAGCGCGCGGCGGGCGGGAGTCGCTGCGTTGCCTTCGCCCCGT
GCCCCGCTCCGCGCCGCCTCGCGCCGCCCGCCCCGGCTCTGACTGACCGCGTTACTCCCACAGGTGAGCGGGCG
GGACGGCCCTTCTCCTCCGGGCTGTAATTAGCGCTTGGTTTAATGACGGCTCGTTTCTTTTCTGTGGCTGCGTGAAA
GCCTTAAAGGGCTCCGGGAGGGCCCTTTGTGCGGGGGGGAGCGGCTCGGGGGGTGCGTGCGTGTGTGTGTGCGT
GGGGAGCGCCGCGTGCGGCCCGCGCTGCCCGGCGGCTGTGAGCGCTGCGGGCGCGGCGCGGGGCTTTGTGCGC
TCCGCGTGTGCGCGAGGGGAGCGCGGCCGGGGGCGGTGCCCCGCGGTGCGGGGGGGCTGCGAGGGGAACAAAG
GCTGCGTGCGGGGTGTGTGCGTGGGGGGGTGAGCAGGGGGTGTGGGCGCGGCGGTCGGGCTGTAACCCCCCCCT
GCACCCCCCTCCCCGAGTTGCTGAGCACGGCCCGGCTTCGGGTGCGGGGCTCCGTGCGGGGCGTGGCGCGGGGC
TCGCCGTGCCGGGCGGGGGGTGGCGGCAGGTGGGGGTGCCGGGCGGGGCGGGGCCGCCTCGGGCCGGGGAGG
GCTCGGGGGAGGGGCGCGGCGGCCCCCGGAGCGCCGGCGGCTGTCGAGGCGCGGCGAGCCGCAGCCATTGCCT
TTTATGGTAATCGTGCGAGAGGGCGCAGGGACTTCCTTTGTCCCAAATCTGTGCGGAGCCGAAATCTGGGAGGCGC
CGCCGCACCCCCTCTAGCGGGCGCGGGGCGAAGCGGTGCGGCGCCGGCAGGAAGGAAATGGGCGGGGAGGGCC
TTCGTGCGTCGCCGCGCCGCCGTCCCCTTCTCCCTCTCCAGCCTCGGGGCTGTCCGCGGGGGGACGGCTGCCTTC
GGGGGGGACGGGGCAGGGCGGGGTTCGGCTTCTGGCGTGTGACCGGCGGCTCTAGCGCCTCTGCTAACCATGTTC
ATGCCTTCTTCTTTTTCCTACAGCTCCTGGGCAACGTGCTGGTTGTTGTGCTGTCTCATCATTTTGGCAAAGAATTGC
GGCCGTCTCAGGCCGAGTTCGGTACGGTACCGCCACCATGGCCATGAGAAGCGGCAGGCATCCTAGCCTGCTGCT
GCTGCTGGTCCTGCTGCTGTGGCTGCTCCAGGTGAGCATTATCGATTCCGTGCAGCAAGAAACCGACGACCTGACA
AAGCAAACCAAAGAAAAAATCTATCAGCCCCTGAGAAGAAGCAAGAGGAGGTGGGTGATCACCACACTGGAACTCGA
GGAAGAAGACCCCGGCCCCTTTCCCAAGCTGATCGGCGAGCTGTTCAACAACATGTCCTATAACATGAGCCTCATGT
ACCTGATTTCCGGCCCTGGCGTCGACGAATACCCTGAGATCGGCCTGTTTAGCCTGGAGGACCACGAGAACGGCAG
GATCTACGTGCACAGGCCTGTGGACAGGGAGATGACCCCCAGCTTCACAGTCTACTTCGACGTGGTGGAGAGGTCC
ACCGGAAAGATCGTGGATACCTCCCTGATTTTTAACATCAGGATTAGCGACGTCAACGACCATGCCCCCCAGTTCCC
TGAGAAGGAGTTCAATATTACCGTCCAGGAGAACCAGAGCGCCGGCCAGCCTATCTTCCAAATGCTCGCCGTGGATC
TCGATGAGGAGAACACCCCTAACTCCCAGGTGCTGTATTTCCTCATTAGCCAGACACCTCTGCTGAAGGAGAGCGGC
TTCAGAGTCGACAGACTGTCCGGAGAAATCAGGCTGAGCGGCTGTCTGGACTATGAGACCGCCCCCCAATTTACCCT
GCTGATTAGGGCCAGAGACTGTGGCGAACCTTCCCTCAGCTCCACCACCACCGTCCACGTCGATGTGCAGGAGGGC
AACAACCATAGGCCTGCCTTTACCCAGGAGAACTACAAGGTGCAAATCCCTGAAGGAAGGGCCTCCCAGGGCGTGC
TCAGACTGCTCGTGCAAGATAGGGACTCCCCCTTTACCAGCGCCTGGAGGGCTAAGTTTAATATCCTGCACGGAAAC
GAGGAGGGCCATTTTGACATCTCCACCGATCCCGAGACCAACGAAGGCATCCTCAATGTGATCAAGCCCCTGGATTA
TGAAACCAGGCCCGCCCAGAGCCTGATCATCGTGGTGGAAAATGAGGAGAGGCTCGTGTTCTGCGAAAGAGGCAAG
CTCCAGCCCCCCAGGAAAGCCGCTGCTAGCGCCACAGTCTCCGTCCAGGTCACCGACGCCAACGATCCTCCCGCCT
TCCACCCCCAATCCTTCATCGTGAACAAAGAGGAGGGCGCCAGACCTGGCACCCTCCTGGGCACCTTTAATGCCAT
GGACCCCGATTCCCAGATTAGGTACGAGCTCGTGCACGACCCTGCCAACTGGGTGTCCGTCGATAAGAACAGCGGA
GTCGTCATTACCGTGGAGCCCATCGACAGGGAATCCCCCCACGTGAACAATTCCTTCTATGTGATCATCATCCACGC
TGTGGACGATGGCTTTCCTCCTCAAACAGCCACCGGCACCCTCATGCTGTTTCTGTCCGACATCAACGACAACGTCC
CTACACTCAGGCCTAGGTCCAGGTACATGGAGGTGTGCGAATCCGCTGTGCACGAGCCTCTCCACATCGAAGCCGA
GGACCCTGACCTCGAGCCCTTCAGCGACCCTTTCACCTTCGAACTGGACAATACCTGGGGAAACGCTGAAGACACAT
GGAAGCTGGGCAGGAACTGGGGCCAGTCCGTGGAACTGCTCACACTCAGGTCCCTGCCTAGGGGCAACTACCTCG
TCCCTCTGTTTATCGGAGACAAGCAGGGACTGTCCCAAAAGCAGACAGTGCACGTGAGGATCTGTCCTTGCGCCAG
CGGCCTCACATGCGTCGAACTGGCCGATGCCGAAGTGGGACTGCACGTGGGCGCTCTGTTTCCCGTCTGTGCCGCT
TTTGTGGCCCTGGCTGTGGCCCTCCTGTTTCTCCTGAGGTGTTACTTCGTGCTGGAACCCAAGAGGCACGGCTGCTC
CGTCAGCAATGATGAAGGCCACCAGACCCTGGTCATGTATAACGCCGAGAGCAAGGGCACCTCCGCCCAGACCTGG
AGCGACGTGGAGGGACAGAGACCTGCCCTGCTGATCTGCACAGCTGCTGCTGGCCCCACACAGGGCGTGAAGGAC
CTGGAGGAAGTGCCCCCTTCCGCTGCTAGCCAGAGCGCCCAGGCCAGATGTGCTCTCGGCTCCTGGGGCTACGGA
AAACCCTTCGAGCCCAGGAGCGTCAAAAACATCCACAGCACCCCCGCCTATCCTGACGCCACCATGCATAGGCAGC
TCCTGGCCCCCGTGGAAGGCAGGATGGCCGAAACCCTGAATCAGAAACTGCACGTCGCTAACGTCCTGGAAGACGA
TCCTGGCTATCTGCCCCACGTGTACTCCGAAGAGGGAGAGTGTGGCGGAGCTCCCTCCCTGTCCTCCCTGGCTTCC

CTGGAGCAGGAACTGCAGCCCGACCTCCTCGACTCCCTGGGAAGCAAGGCCACCCCCTTCGAGGAGATCTATAGCG
AGTCCGGCGTGCCTTCCGGATCCTCCGCTTGGAGCCACCCCCAGTTTGAAAAAGGCGGCGGCTCCGGCGGCGGCT
CCGGCGGAAGCGCCTGGTCCCATCCCCAGTTCGAAAAGTAGTAAGAATTCATTCCATCACCACCATCATCACCATCA
TTGACTCGAGCGATAATTCACTCCTCAGGTGCAGGCTGCCTATCAGAAGGTGGTGGCTGGTGTGGCCAATGCCCTG
GCTCACAAATACCACTGAGATCTTTTTCCCTCTGCCAAAAATTATGGGGACATCATGAAGCCCCTTGAGCATCTGACT
TCTGGCTAATAAAGGAAATTTATTTTCATTGCAATAGTGTGTTGGAATTTTTTGTGTCTCTCACTCGGAAGGACATATG
GGAGGGCAAATCATTTAAAACATCAGAATGAGTATTTGGTTTAGAGTTTGGCAACATATGCCATATGCTGGCTGCCAT
GAACAAAGGTGGCTATAAAGAGGTCATCAGTATATGAAACAGCCCCCTGCTGTCCATTCCTTATTCCATAGAAAAGCC
TTGACTTGAGGTTAGATTTTTTTTATATTTTGTTTTGTGTTATTTTTTTCTTTAACATCCCTAAAATTTTCCTTACATGTTT
TACTAGCCAGATTTTTCCTCCTCTCCTGACTACTCCCAGTCATAGCTGTCCCTCTTCTCTTATGAAGATCCCTCGACCT
GCAGCCCAAGCTTGGCGTAATCATGGTCATAGCTGTTTCCTGTGTGAAATTGTTATCCGCTCACAATTCCACACAACA
TACGAGCCGGAAGCATAAAGTGTAAAGCCTGGGGTGCCTAATGAGTGAGCTAACTCACATTAATTGCGTTGCGCTCA
CTGCCCGCTTTCCAGTCGGGAAACCTGTCGTGCCAGCGGATCGATCCGCTGCATTAATGAATCGGCCAACGCGCGG
GGAGAGGCGGTTTGCGTATTGGGCGCTCTTCCGCTTCCTCGCTCACTGACTCGCTGCGCTCGGTCGTTCGGCTGCG
GCGAGCGGTATCAGCTCACTCAAAGGCGGTAATACGGTTATCCACAGAATCAGGGGATAACGCAGGAAAGAACATGT
GAGCAAAAGGCCAGCAAAAGGCCAGGAACCGTAAAAAGGCCGCGTTGCTGGCGTTTTTCCATAGGCTCCGCCCCCC
TGACGAGCATCACAAAAATCGACGCTCAAGTCAGAGGTGGCGAAACCCGACAGGACTATAAAGATACCAGGCGTTTC
CCCCTGGAAGCTCCCTCGTGCGCTCTCCTGTTCCGACCCTGCCGCTTACCGGATACCTGTCCGCCTTTCTCCCTTCG
GGAAGCGTGGCGCTTTCTCAATGCTCACGCTGTAGGTATCTCAGTTCGGTGTAGGTCGTTCGCTCCAAGCTGGGCTG
TGTGCACGAACCCCCCGTTCAGCCCGACCGCTGCGCCTTATCCGGTAACTATCGTCTTGAGTCCAACCCGGTAAGAC
ACGACTTATCGCCACTGGCAGCAGCCACTGGTAACAGGATTAGCAGAGCGAGGTATGTAGGCGGTGCTACAGAGTT
CTTGAAGTGGTGGCCTAACTACGGCTACACTAGAAGGACAGTATTTGGTATCTGCGCTCTGCTGAAGCCAGTTACCTT
CGGAAAAAGAGTTGGTAGCTCTTGATCCGGCAAACAAACCACCGCTGGTAGCGGTGGTTTTTTTGTTTGCAAGCAGC
AGATTACGCGCAGAAAAAAAGGATCTCAAGAAGATCCTTTGATCTTTTCTACGGGGTCTGACGCTCAGTGGAACGAAA
ACTCACGTTAAGGGATTTTGGTCATGAGATTATCAAAAAGGATCTTCACCTAGATCCTTTTAAATTAAAAATGAAGTTTT
AAATCAATCTAAAGTATATATGAGTAAACTTGGTCTGACAGTTACCAATGCTTAATCAGTGAGGCACCTATCTCAGCGA
TCTGTCTATTTCGTTCATCCATAGTTGCCTGACTCCCCGTCGTGTAGATAACTACGATACGGGAGGGCTTACCATCTG
GCCCCAGTGCTGCAATGATACCGCGAGACCCACGCTCACCGGCTCCAGATTTATCAGCAATAAACCAGCCAGCCGG
AAGGGCCGAGCGCAGAAGTGGTCCTGCAACTTTATCCGCCTCCATCCAGTCTATTAATTGTTGCCGGGAAGCTAGAG
TAAGTAGTTCGCCAGTTAATAGTTTGCGCAACGTTGTTGCCATTGCTACAGGCATCGTGGTGTCACGCTCGTCGTTTG
GTATGGCTTCATTCAGCTCCGGTTCCCAACGATCAAGGCGAGTTACATGATCCCCCATGTTGTGCAAAAAAGCGGTT
AGCTCCTTCGGTCCTCCGATCGTTGTCAGAAGTAAGTTGGCCGCAGTGTTATCACTCATGGTTATGGCAGCACTGCA
TAATTCTCTTACTGTCATGCCATCCGTAAGATGCTTTTCTGTGACTGGTGAGTACTCAACCAAGTCATTCTGAGAATAG
TGTATGCGGCGACCGAGTTGCTCTTGCCCGGCGTCAATACGGGATAATACCGCGCCACATAGCAGAACTTTAAAAGT
GCTCATCATTGGAAAACGTTCTTCGGGGCGAAAACTCTCAAGGATCTTACCGCTGTTGAGATCCAGTTCGATGTAACC
CACTCGTGCACCCAACTGATCTTCAGCATCTTTTACTTTCACCAGCGTTTCTGGGTGAGCAAAAACAGGAAGGCAAAA
TGCCGCAAAAAAGGGAATAAGGGCGACACGGAAATGTTGAATACTCATACTCTTCCTTTTTCAATATTATTGAAGCATT
TATCAGGGTTATTGTCTCATGAGCGGATACATATTTGAATGTATTTAGAAAAATAAACAAATAGGGGTTCCGCGCACAT
TTCCCCGAAAAGTGCCACCTG

> cmv6_CDH26-AC-mGFP 9128 bp SgfI (AsiSI) highlighted in Yellow MluI highlighted in Pink Inserted Sequence in Blue

AACAAAATATTAACGCTTACAATTTCCATTCGCCATTCAGGCTGCGCAACTGTTGGGAAGGGCGATCGGTGCGGGCC
TCTTCGCTATTACGCCAGCTGGCGAAAGGGGGATGTGCTGCAAGGCGATTAAGTTGGGTAACGCCAGGGTTTTCCC
AGTCACGACGTTGTAAAACGACGGCCAGTGCCAAGCTGATCTATACATTGAATCAATATTGGCAATTAGCCATATTAG
TCATTGGTTATATAGCATAAATCAATATTGGCTATTGGCCATTGCATACGTTGTATCTATATCATAATATGTACATTTATA
TTGGCTCATGTCCAATATGACCGCCATGTTGACATTGATTATTGACTAGTTATTAATAGTAATCAATTACGGGGTCATT
AGTTCATAGCCCATATATGGAGTTCCGCGTTACATAACTTACGGTAAATGGCCCGCCTGGCTGACCGCCCAACGACC
CCCGCCCATTGACGTCAATAATGACGTATGTTCCCATAGTAACGCCAATAGGGACTTTCCATTGACGTCAATGGGTG
GAGTATTTACGGTAAACTGCCCACTTGGCAGTACATCAAGTGTATCATATGCCAAGTCCGCCCCCTATTGACGTCAAT
GACGGTAAATGGCCCGCCTGGCATTATGCCCAGTACATGACCTTACGGGACTTTCCTACTTGGCAGTACATCTACGT
ATTAGTCATCGCTATTACCATGGTGATGCGGTTTTGGCAGTACACCAATGGGCGTGGATAGCGGTTTGACTCACGGG
GATTTCCAAGTCTCCACCCCATTGACGTCAATGGGAGTTTGTTTTGGCACCAAAATCAACGGGACTTTCCAAAATGTC
GTAATAACCCCGCCCCGTTGACGCAAATGGGCGGTAGGCGTGTACGGTGGGAGGTCTATATAAGCAGAGCTCGTTT
AGTGAACCGTCAGAATTTTGTAATACGACTCACTATAGGGCGGCCGGGAATTCGTCGACTGGATCCGGTACCGAGGA
GATGCGATCGCCATGGCCATGAGATCCGGGAGGCACCCCTCGCTGCTGCTGCTTCTAGTGCTGCTGCTGTGGCTGC
TGCAGGTCAGTATCATTGACAGTGTTCAACAGGAAACAGATGATCTTACTAAGCAAACAAAGGAAAAGATCTACCAGC
CTCTACGGCGATCCAAGAGAAGATGGGTTATCACCACCTTGGAGCTGGAGGAGGAAGACCCGGGACCCTTTCCCAA
ACTCATTGGTGAGCTGTTCAATAATATGTCTTATAACATGTCACTAATGTATCTAATCAGTGGACCTGGTGTGGATGAA
TATCCAGAGATTGGTTTGTTTTCTCTAGAAGATCATGAGAACGGAAGGATATATGTTCACCGCCCTGTCGATCGAGAA
ATGACACCATCTTTCACGGTTTATTTTGATGTTGTGGAGCGCTCAACAGGAAAAATTGTGGATACATCCTTGATTTTCA
ACATTAGGATCAGTGATGTGAATGATCATGCACCCCAGTTTCCAGAGAAGGAATTTAACATCACTGTGCAAGAAAACC
AATCTGCAGGGCAACCTATTTTTCAGATGTTAGCAGTCGATTTGGATGAAGAAAACACTCCAAATTCTCAAGTCCTTTA
CTTCCTCATTTCTCAAACACCATTACTGAAAGAAAGTGGTTTCCGGGTTGATCGCCTTAGTGGAGAAATACGACTCTC
TGGCTGCTTAGATTATGAGACCGCTCCTCAGTTTACACTGCTAATCAGAGCCAGGGACTGTGGAGAACCGTCACTGT
CATCCACGACCACCGTTCACGTGGATGTGCAAGAAGGCAACAACCCACAGGCCTGCATTTACCCAGGAGAACTATAAG
GTTCAGATTCCTGAAGGCCGAGCCAGCCAGGGCGTGTTGCGTCTCCTGGTTCAAGATCGAGATTCTCCATTTACATC
AGCTTGGAGAGCAAAATTCAACATATTGCATGGCAATGAAGAGGGGCATTTTGACATTTCGACTGACCCTGAGACCAA
CGAAGGGATATTAAATGTTATCAAGCCTTTGGATTATGAGACTCGCCCAGCGCAAAGCCTCATCATTGTCGTGGAGAA
TGAGGAGAGGCTCGTCTTCTGTGAGAGAGGAAAGCTTCAGCCGCCAAGGAAGGCAGCAGCCAGCGCCACTGTGAG
TGTGCAGGTGACAGACGCCAACGACCCACCAGCCTTTCACCCCCAGAGCTTCATTGTCAATAAAGAGGAGGGCGCC
AGGCCTGGGACCCTGTTGGGAACTTTTAATGCCATGGATCCAGACAGCCAGATAAGATATGAACTGGTTCATGACCC
AGCAAATTGGGTCAGCGTCGACAAAAACTCCGGAGTGGTCATCACCGTGGAGCCAATTGACCGAGAATCCCCTCATG
TAAATAACAGTTTTTATGTAATCATCATTCACGCTGTTGATGATGGCTTCCCACCGCAGACTGCTACAGGGACCCTAAT
GCTCTTCCTGTCTGACATCAATGACAACGTCCCGACTCTCCGGCCACGTTCCCGCTACATGGAGGTCTGTGAGTCTG
CTGTGCATGAGCCCCTCCACATCGAGGCAGAGGATCCGGACCTGGAGCCGTTCTCTGACCCATTTACATTTGAATTG
GACAATACCTGGGGAAATGCGGAGGACACATGGAAGTTGGGGAGAAATTGGGGTCAATCAGTTGAACTTTTAACCTT
GAGAAGCCTGCCACGTGGTAATTACTTGGTGCCACTCTTCATTGGAGACAAACAGGGACTTTCCCAGAAGCAAACTG
TCCATGTAAGGATCTGCCCCTGTGCCAGTGGGCTCACATGTGTGGAGCTTGCAGATGCAGAAGTGGGGCTTCATGT
GGGGGCCCTGTTCCCTGTCTGTGCAGCATTTGTGGCTCTGGCAGTGGCTCTGCTTTTTCTGTTGCGATGCTATTTTGT
GCTTGAACCTAAGAGGCATGGATGCTCTGTATCCAATGATGAAGGCCACCAAACACTGGTCATGTATAATGCGGAGA
GCAAAGGCACTTCAGCCCAGACATGGTCAGATGTTGAAGGCCAGAGGCCGGCTCTGCTCATCTGCACAGCTGCAGC
AGGACCCACGCAGGGAGTTAAGGATCTCGAGGAAGTGCCTCCATCTGCAGCGAGTCAGTCAGCCCAAGCACGCTGT
GCTCTGGGGAGCTGGGGTTATGGCAAGCCCTTTGAGCCAAGAAGTGTGAAAAACATACACTCTACTCCTGCTTACCC
AGATGCCACAATGCACAGACAACTCCTGGCTCCGGTGGAAGGAAGGATGGCAGAGACATTGAATCAGAAACTCCAT
GTTGCCAATGTGCTGGAAGATGACCCCGGCTACCTACCTCACGTCTACAGCGAGGAAGGGGAGTGTGGAGGGGCC
CCATCCCTCAGCTCTCTGGCCAGCTTGGAACAGGAGTTGCAACCTGATTTGCTGGACTCTTTGGGTTCAAAAGCGAC
TCCGTTTGAGGAAATATATTCAGAGTCAGGTGTTCCTTCCCTGCCGCCCGGCGCGCCAGATCTCAAGCTTAACTAGTT
AGCGGACCGACGCGTACGCGGCCGCTCGAGATGAGCGGGGGCGAGGAGCTGTTCGCCGGCATCGTGCCCGTGCT
GATCGAGCTGGACGGCGACGTGCACGGCCACAAGTTCAGCGTGCGCGGCGAGGGCGAGGGCGACGCCGACTACG
GCAAGCTGGAGATCAAGTTCATCTGCACCACCGGCAAGCTGCCCGTGCCCTGGCCCACCCTGGTGACCACCCTCTG
CTACGGCATCCAGTGCTTCGCCCGCTACCCCGAGCACATGAAGATGAACGACTTCTTCAAGAGCGCCATGCCCGAG
GGCTACATCCAGGAGCGCACCATCCAGTTCCAGGACGACGGCAAGTACAAGACCCGCGGCGAGGTGAAGTTCGAG
GGCGACACCCTGGTGAACCGCATCGAGCTGAAGGGCAAGGACTTCAAGGAGGACGGCAACATCCTGGGCCACAAG
CTGGAGTACAGCTTCAACAGCCACAACGTGTACATCCGCCCCGACAAGGCCAACAACGGCCTGGAGGCTAACTTCA
AGACCCGCCACAACATCGAGGGCGGCGGCGTGCAGCTGGCCGACCACTACCAGACCAACGTGCCCCTGGGCGACG
GCCCCGTGCTGATCCCCATCAACCACTACCTGAGCACTCAGACCAAGATCAGCAAGGACCGCAACGAGGCCCGCGA
CCACATGGTGCTCCTGGAGTCCTTCAGCGCCTGCTGCCACACCCACGGCATGGACGAGCTGTACAGGTCCGGACTC
AGATAAGTTTAAACGGCCGGCCGCGGTCATAGCTGTTTCCTGAACAGATCCCGGGTGGCATCCCTGTGACCCCTCCC
CAGTGCCTCTCCTGGCCCTGGAAGTTGCCACTCCAGTGCCCACCAGCCTTGTCCTAATAAAATTAAGTTGCATCATTT

TGTCTGACTAGGTGTCCTTCTATAATATTATGGGGTGGAGGGGGGTGGTATGGAGCAAGGGGCAAGTTGGGAAGAC
AACCTGTAGGGCCTGCGGGGTCTATTGGGAACCAAGCTGGAGTGCAGTGGCACAATCTTGGCTCACTGCAATCTCC
GCCTCCTGGGTTCAAGCGATTCTCCTGCCTCAGCCTCCCGAGTTGTTGGGATTCCAGGCATGCATGACCAGGCTCAG
CTAATTTTTGTTTTTTTGGTAGAGACGGGGTTTCACCATATTGGCCAGGCTGGTCTCCAACTCCTAATCTCAGGTGATC
TACCCACCTTGGCCTCCCAAATTGCTGGGATTACAGGCGTGAACCACTGCTCCCTTCCCTGTCCTTCTGATTTTAAAA
TAACTATACCAGCAGGAGGACGTCCAGACACAGCATAGGCTACCTGGCCATGCCCAACCGGTGGGACATTTGAGTT
GCTTGCTTGGCACTGTCCTCTCATGCGTTGGGTCCACTCAGTAGATGCCTGTTGAATTGGGTACGCGGCCAGCTTGG
CTGTGGAATGTGTGTCAGTTAGGGTGTGGAAAGTCCCCAGGCTCCCCAGCAGGCAGAAGTATGCAAAGCATGCATC
TCAATTAGTCAGCAACCAGGTGTGGAAAGTCCCCAGGCTCCCCAGCAGGCAGAAGTATGCAAAGCATGCATCTCAAT
TAGTCAGCAACCATAGTCCCGCCCCTAACTCCGCCCATCCCGCCCCTAACTCCGCCCAGTTCCGCCCATTCTCCGCC
CCATGGCTGACTAATTTTTTTTATTTATGCAGAGGCCGAGGCCGCCTCGGCCTCTGAGCTATTCCAGAAGTAGTGAG
GAGGCTTTTTTGGAGGCCTAGGCTTTTGCAAAAAGCTCCCGGGAGCTTGTATATCCATTTTCGGATCTGATCAAGAGA
CAGGATGAGGATCGTTTCGCATGATTGAACAAGATGGATTGCACGCAGGTTCTCCGGCCGCTTGGGTGGAGAGGCT
ATTCGGCTATGACTGGGCACAACAGACAATCGGCTGCTCTGATGCCGCCGTGTTCCGGCTGTCAGCGCAGGGGCGC
CCGGTTCTTTTTGTCAAGACCGACCTGTCCGGTGCCCTGAATGAACTGCAGGACGAGGCAGCGCGGCTATCGTGGC
TGGCCACGACGGGCGTTCCTTGCGCAGCTGTGCTCGACGTTGTCACTGAAGCGGGAAGGGACTGGCTGCTATTGGG
CGAAGTGCCGGGGCAGGATCTCCTGTCATCTCACCTTGCTCCTGCCGAGAAAGTATCCATCATGGCTGATGCAATGC
GGCGGCTGCATACGCTTGATCCGGCTACCTGCCCATTCGACCACCAAGCGAAACATCGCATCGAGCGAGCACGTAC
TCGGATGGAAGCCGGTCTTGTCGATCAGGATGATCTGGACGAAGAGCATCAGGGGCTCGCGCCAGCCGAACTGTTC
GCCAGGCTCAAGGCGCGCATGCCCGACGGCGAGGATCTCGTCGTGACCCATGGCGATGCCTGCTTGCCGAATATC
ATGGTGGAAAATGGCCGCTTTTCTGGATTCATCGACTGTGGCCGGCTGGGTGTGGCCGACCGCTATCAGGACATAG
CGTTGGCTACCCGTGATATTGCTGAAGAGCTTGGCGGCGAATGGGCTGACCGCTTCCTCGTGCTTTACGGTATCGCC
GCTCCCGATTCGCAGCGCATCGCCTTCTATCGCCTTCTTGACGAGTTCTTCTGAGCGGGACTCTGGGGTTCGAAATG
ACCGACCAAGCGACGCCCAACCTGCCATCACGAGATTTCGATTCCACCGCCGCCTTCTATGAAAGGTTGGGCTTCG
GAATCGTTTTCCGGGACGCCGGCTGGATGATCCTCCAGCGCGGGGATCTCATGCTGGAGTTCTTCGCCCACCCCAA
CTTGTTTATTGCAGCTTATAATGGTTACAAATAAAGCAATAGCATCACAAATTTCACAAATAAAGCATTTTTTTCACTGC
ATTCTAGTTGTGGTTTGTCCAAACTCATCAATGTATCTTATCATGTCTGTATACCGTCGACCTCTAGCTAGAGCTTGGC
GTAATCATGGTCATAGCTGTTTCCTGTGTGAAATTGTTATCCGCTCACAATTCCACACAACATACGAGCCGGAAGCAT
AAAGTGTAAAGCCTGGGGTGCCTAATGAGTGAGCTAACTCACATTAATTGCGTTGCGCTCACTGCCCGCTTTCCAGT
CGGGAAACCTGTCGTGCCAGCTGCATTAATGAATCGGCCAACGCGCGGGGAGAGGCGGTTTGCGTATTGGGCGCT
CTTCCGCTTCCTCGCTCACTGACTCGCTGCGCTCGGTCGTTCGGCTGCGGCGAGCGGTATCAGCTCACTCAAAGGC
GGTAATACGGTTATCCACAGAATCAGGGGATAACGCAGGAAAGAACATGTGAGCAAAAGGCCAGCAAAAGGCCAGG
AACCGTAAAAAGGCCGCGTTGCTGGCGTTTTTCCATAGGCTCCGCCCCCCTGACGAGCATCACAAAAATCGACGCTC
AAGTCAGAGGTGGCGAAACCCGACAGGACTATAAAGATACCAGGCGTTTCCCCCTGGAAGCTCCCTCGTGCGCTCT
CCTGTTCCGACCCTGCCGCTTACCGGATACCTGTCCGCCTTTCTCCCTTCGGGAAGCGTGGCGCTTTCTCATAGCTC
ACGCTGTAGGTATCTCAGTTCGGTGTAGGTCGTTCGCTCCAAGCTGGGCTGTGTGCACGAACCCCCCGTTCAGCCC
GACCGCTGCGCCTTATCCGGTAACTATCGTCTTGAGTCCAACCCGGTAAGACACGACTTATCGCCACTGGCAGCAGC
CACTGGTAACAGGATTAGCAGAGCGAGGTATGTAGGCGGTGCTACAGAGTTCTTGAAGTGGTGGCCTAACTACGGC
TACACTAGAAGAACAGTATTTGGTATCTGCGCTCTGCTGAAGCCAGTTACCTTCGGAAAAAGAGTTGGTAGCTCTTGA
TCCGGCAAACAAACCACCGCTGGTAGCGGTGGTTTTTTTGTTTGCAAGCAGCAGATTACGCGCAGAAAAAAAGGATC
TCAAGAAGATCCTTTGATCTTTTCTACGGGGTCTGACGCTCAGTGGAACGAAAACTCACGTTAAGGGATTTTGGTCAT
GAGATTATCAAAAAGGATCTTCACCTAGATCCTTTTAAATTAAAAATGAAGTTTTAAATCAATCTAAAGTATATATGAGT
AAACTTGGTCTGACAGTTACCAATGCTTAATCAGTGAGGCACCTATCTCAGCGATCTGTCTATTTCGTTCATCCATAGT
TGCCTGACTCCCCGTCGTGTAGATAACTACGATACGGGAGGGCTTACCATCTGGCCCCAGTGCTGCAATGATACCGC
GAGACCCACGCTCACCGGCTCCAGATTTATCAGCAATAAACCAGCCAGCCGGAAGGGCCGAGCGCAGAAGTGGTCC
TGCAACTTTATCCGCCTCCATCCAGTCTATTAATTGTTGCCGGGAAGCTAGAGTAAGTAGTTCGCCAGTTAATAGTTT
GCGCAACGTTGTTGCCATTGCTACAGGCATCGTGGTGTCACGCTCGTCGTTTGGTATGGCTTCATTCAGCTCCGGTT
CCCAACGATCAAGGCGAGTTACATGATCCCCCATGTTGTGCAAAAAAGCGGTTAGCTCCTTCGGTCCTCCGATCGTT
GTCAGAAGTAAGTTGGCCGCAGTGTTATCACTCATGGTTATGGCAGCACTGCATAATTCTCTTACTGTCATGCCATCC
GTAAGATGCTTTTCTGTGACTGGTGAGTACTCAACCAAGTCATTCTGAGAATAGTGTATGCGGCGACCGAGTTGCTCT
TGCCCGGCGTCAATACGGGATAATACCGCGCCACATAGCAGAACTTTAAAAGTGCTCATCATTGGAAAACGTTCTTC
GGGGCGAAAACTCTCAAGGATCTTACCGCTGTTGAGATCCAGTTCGATGTAACCCACTCGTGCACCCAACTGATCTT
CAGCATCTTTTACTTTCACCAGCGTTTCTGGGTGAGCAAAAACAGGAAGGCAAAATGCCGCAAAAAAGGGAATAAGG
GCGACACGGAAATGTTGAATACTCATACTCTTCCTTTTTCAATATTATTGAAGCATTTATCAGGGTTATTGTCTCATGAG
CGGATACATATTTGAATGTATTTAGAAAAATAAACAAATAGGGGTTCCGCGCACATTTCCCCGAAAAGTGCCACCTGA
CGCGCCCTGTAGCGGCGCATTAAGCGCGGCGGGTGTGGTGGTTACGCGCAGCGTGACCGCTACACTTGCCAGCGC
CCTAGCGCCCGCTCCTTTCGCTTTCTTCCCTTCCTTTCTCGCCACGTTCGCCGGCTTTCCCCGTCAAGCTCTAAATCG
GGGGCTCCCTTTAGGGTTCCGATTTAGTGCTTTACGGCACCTCGACCCCAAAAAACTTGATTAGGGTGATGGTTCAC
GTAGTGGGCCATCGCCCTGATAGACGGTTTTTCGCCCTTTGACGTTGGAGTCCACGTTCTTTAATAGTGGACTCTTGT

TCCAAACTGGAACAACACTCAACCCTATCTCGGTCTATTCTTTTGATTTATAAGGGATTTTGCCGATTTCGGCCTATTG
GTTAAAAAATGAGCTGATTTAACAAAAATTTAACGCGAATTTT

>pLL3.7 LifAct-mCherry 7690 bp NheI highlighted in Yellow EcoRI highlighted in Pink Inserted Sequence in Blue

GTCGACGGATCGGGAGATCTCCCGATCCCCTATGGTGCACTCTCAGTACAATCTGCTCTGATGCCGCATAGTTAAGC
CAGTATCTGCTCCCTGCTTGTGTGTTGGAGGTCGCTGAGTAGTGCGCGAGCAAAATTTAAGCTACAACAAGGCAAGG
CTTGACCGACAATTGCATGAAGAATCTGCTTAGGGTTAGGCGTTTTGCGCTGCTTCGCGATGTACGGGCCAGATATA
CGCGTTGACATTGATTATTGACTAGTTATTAATAGTAATCAATTACGGGGTCATTAGTTCATAGCCCATATATGGAGTT
CCGCGTTACATAACTTACGGTAAATGGCCCGCCTGGCTGACCGCCCAACGACCCCCGCCCATTGACGTCAATAATGA
CGTATGTTCCCATAGTAACGCCAATAGGGACTTTCCATTGACGTCAATGGGTGGAGTATTTACGGTAAACTGCCCACT
TGGCAGTACATCAAGTGTATCATATGCCAAGTACGCCCCCTATTGACGTCAATGACGGTAAATGGCCCGCCTGGCAT
TATGCCCAGTACATGACCTTATGGGACTTTCCTACTTGGCAGTACATCTACGTATTAGTCATCGCTATTACCATGGTGA
TGCGGTTTTGGCAGTACATCAATGGGCGTGGATAGCGGTTTGACTCACGGGGATTTCCAAGTCTCCACCCCATTGAC
GTCAATGGGAGTTTGTTTTGGCACCAAAATCAACGGGACTTTCCAAAATGTCGTAACAACTCCGCCCCATTGACGCAA
ATGGGCGGTAGGCGTGTACGGTGGGAGGTCTATATAAGCAGCGCGTTTTGCCTGTACTGGGTCTCTCTGGTTAGAC
CAGATCTGAGCCTGGGAGCTCTCTGGCTAACTAGGGAACCCACTGCTTAAGCCTCAATAAAGCTTGCCTTGAGTGCT
TCAAGTAGTGTGTGCCCGTCTGTTGTGTGACTCTGGTAACTAGAGATCCCTCAGACCCTTTTAGTCAGTGTGGAAAAT
CTCTAGCAGTGGCGCCCGAACAGGGACTTGAAAGCGAAAGGGAAACCAGAGGAGCTCTCTCGACGCAGGACTCGG
CTTGCTGAAGCGCGCACGGCAAGAGGCGAGGGGCGGCGACTGGTGAGTACGCCAAAAATTTTGACTAGCGGAGGC
TAGAAGGAGAGAGATGGGTGCGAGAGCGTCAGTATTAAGCGGGGGAGAATTAGATCGCGATGGGAAAAAATTCGGT
TAAGGCCAGGGGGAAAGAAAAAATATAAATTAAAACATATAGTATGGGCAAGCAGGGAGCTAGAACGATTCGCAGTT
AATCCTGGCCTGTTAGAAACATCAGAAGGCTGTAGACAAATACTGGGACAGCTACAACCATCCCTTCAGACAGGATC
AGAAGAACTTAGATCATTATATAATACAGTAGCAACCCTCTATTGTGTGCATCAAAGGATAGAGATAAAAGACACCAAG
GAAGCTTTAGACAAGATAGAGGAAGAGCAAAACAAAAGTAAGACCACCGCACAGCAAGCGGCCGGCCGCGCTGATC
TTCAGACCTGGAGGAGGAGATATGAGGGACAATTGGAGAAGTGAATTATATAAATATAAAGTAGTAAAAATTGAACCA
TTAGGAGTAGCACCCACCAAGGCAAAGAGAAGAGTGGTGCAGAGAGAAAAAAGAGCAGTGGGAATAGGAGCTTTGT
TCCTTGGGTTCTTGGGAGCAGCAGGAAGCACTATGGGCGCAGCGTCAATGACGCTGACGGTACAGGCCAGACAATT
ATTGTCTGGTATAGTGCAGCAGCAGAACAATTTGCTGAGGGCTATTGAGGCGCAACAGCATCTGTTGCAACTCACAG
TCTGGGGCATCAAGCAGCTCCAGGCAAGAATCCTGGCTGTGGAAAGATACCTAAAGGATCAACAGCTCCTGGGGATT
TGGGGTTGCTCTGGAAAACTCATTTGCACCACTGCTGTGCCTTGGAATGCTAGTTGGAGTAATAAATCTCTGGAACAG
ATTTGGAATCACACGACCTGGATGGAGTGGGACAGAGAAATTAACAATTACACAAGCTTAATACACTCCTTAATTGAA
GAATCGCAAAACCAGCAAGAAAAGAATGAACAAGAATTATTGGAATTAGATAAATGGGCAAGTTTGTGGAATTGGTTT
AACATAACAAATTGGCTGTGGTATATAAAATTATTCATAATGATAGTAGGAGGCTTGGTAGGTTTAAGAATAGTTTTTG
CTGTACTTTCTATAGTGAATAGAGTTAGGCAGGGATATTCACCATTATCGTTTCAGACCCACCTCCCAACCCCGAGGG
GACCCGACAGGCCCGAAGGAATAGAAGAAGAAGGTGGAGAGAGAGACAGAGACAGATCCATTCGATTAGTGAACGG
ATCGGCACTGCGTGCGCCAATTCTGCAGACAAATGGCAGTATTCATCCACAATTTTAAAAGAAAAGGGGGGATTGGG
GGGTACAGTGCAGGGGAAAGAATAGTAGACATAATAGCAACAGACATACAAACTAAAGAATTACAAAAACAAATTACA
AAAATTCAAAATTTTCGGGTTTATTACAGGGACAGCAGAGATCCAGTTTGGTTAGTACCGGGCCCGCTCTAGAGATCC
GACGCGCCATCTCTAGGCCCGCGCCGGCCCCCTCGCACAGACTTGTGGGAGAAGCTCGGCTACTCCCCTGCCCCG
GTTAATTTGCATATAATATTTCCTAGTAACTATAGAGGCTTAATGTGCGATAAAAGACAGATAATCTGTTCTTTTTAATA
CTAGCTACATTTTACATGATAGGCTTGGATTTCTATAAGAGATACAAATACTAAATTATTATTTTAAAAAACAGCACAAA
AGGAAACTCACCCTAACTGTAAAGTAATTGTGTGTTTTGAGACTATAAATATCCCTTGGAGAAAAGCCTTGTTAACGCG
CGGTGACCCTCGAGGTCGACGGTATCGATAAGCTCGCTTCACGAGATTCCAGCAGGTCGAGGGACCTAATAACTTC
GTATAGCATACATTATACGAAGTTATATTAAGGGTTCCAAGCTTAAGCGGCCGCGTGGATAACCGTATTACCGCCATG
CATTAGTTATTAATAGTAATCAATTACGGGGTCATTAGTTCATAGCCCATATATGGAGTTCCGCGTTACATAACTTACG
GTAAATGGCCCGCCTGGCTGACCGCCCAACGACCCCCGCCCATTGACGTCAATAATGACGTATGTTCCCATAGTAAC
GCCAATAGGGACTTTCCATTGACGTCAATGGGTGGAGTATTTACGGTAAACTGCCCACTTGGCAGTACATCAAGTGTA
TCATATGCCAAGTACGCCCCCTATTGACGTCAATGACGGTAAATGGCCCGCCTGGCATTATGCCCAGTACATGACCT
TATGGGACTTTCCTACTTGGCAGTACATCTACGTATTAGTCATCGCTATTACCATGGTGATGCGGTTTTGGCAGTACAT
CAATGGGCGTGGATAGCGGTTTGACTCACGGGGATTTCCAAGTCTCCACCCCATTGACGTCAATGGGAGTTTGTTTT
GGCACCAAAATCAACGGGACTTTCCAAAATGTCGTAACAACTCCGCCCCATTGACGCAAATGGGCGGTAGGCGTGTA
CGGTGGGAGGTCTATATAAGCAGAGCTGGTTTAGTGAACCGTCAGATCCGCTAGCGCTACCGGTCGCCACCATGAT
GGGCGTGGCCGACCTGATCAAGAAGTTCGAGAGCATCAGCAAGGAGGAGGTGAGCAAGGGCGAGGAGGATAACAT
GGCCATCATCAAGGAGTTCATGCGCTTCAAGGTGCACATGGAGGGCTCCGTGAACGGCCACGAGTTCGAGATCGAG
GGCGAGGGCGAGGGCCGCCCCTACGAGGGCACCCAGACCGCCAAGCTGAAGGTGACCAAGGGTGGCCCCCTGCC
CTTCGCCTGGGACATCCTGTCCCCTCAGTTCATGTACGGCTCCAAGGCCTACGTGAAGCACCCCGCCGACATCCCC
GACTACTTGAAGCTGTCCTTCCCCGAGGGCTTCAAGTGGGAGCGCGTGATGAACTTCGAGGACGGCGGCGTGGTGA
CCGTGACCCAGGACTCCTCCCTCCAGGACGGCGAGTTCATCTACAAGGTGAAGCTGCGCGGCACCAACTTCCCCTC
CGACGGCCCCGTAATGCAGAAGAAGACCATGGGCTGGGAGGCCTCCTCCGAGCGGATGTACCCCGAGGACGGCGC
CCTGAAGGGCGAGATCAAGCAGAGGCTGAAGCTGAAGGACGGCGGCCACTACGACGCTGAGGTCAAGACCACCTA
CAAGGCCAAGAAGCCCGTGCAGCTGCCCGGCGCCTACAACGTCAACATCAAGTTGGACATCACCTCCCACAACGAG
GACTACACCATCGTGGAACAGTACGAACGCGCCGAGGGCCGCCACTCCACCGGCGGCATGGACGAGCTGTACAAT

AGGAATTCGTCGAGGGACCTAATAACTTCGTATAGCATACATTATACGAAGTTATACATGTTTAAGGGTTCCGGTTCC
ACTAGGTACAATTCGATATCAAGCTTATCGATAATCAACCTCTGGATTACAAAATTTGTGAAAGATTGACTGGTATTCT
TAACTATGTTGCTCCTTTTACGCTATGTGGATACGCTGCTTTAATGCCTTTGTATCATGCTATTGCTTCCCGTATGGCT
TTCATTTTCTCCTCCTTGTATAAATCCTGGTTGCTGTCTCTTTATGAGGAGTTGTGGCCCGTTGTCAGGCAACGTGGC
GTGGTGTGCACTGTGTTTGCTGACGCAACCCCCACTGGTTGGGGCATTGCCACCACCTGTCAGCTCCTTTCCGGGA
CTTTCGCTTTCCCCCTCCCTATTGCCACGGCGGAACTCATCGCCGCCTGCCTTGCCCGCTGCTGGACAGGGGCTCG
GCTGTTGGGCACTGACAATTCCGTGGTGTTGTCGGGGAAATCATCGTCCTTTCCTTGGCTGCTCGCCTGTGTTGCCA
CCTGGATTCTGCGCGGGACGTCCTTCTGCTACGTCCCTTCGGCCCTCAATCCAGCGGACCTTCCTTCCCGCGGCCT
GCTGCCGGCTCTGCGGCCTCTTCCGCGTCTTCGCCTTCGCCCTCAGACGAGTCGGATCTCCCTTTGGGCCGCCTCC
CCGCATCGATACCGTCGACCTCGATCGAGACCTAGAAAAACATGGAGCAATCACAAGTAGCAATACAGCAGCTACCA
ATGCTGATTGTGCCTGGCTAGAAGCACAAGAGGAGGAGGAGGTGGGTTTTCCAGTCACACCTCAGGTACCTTTAAGA
CCAATGACTTACAAGGCAGCTGTAGATCTTAGCCACTTTTTAAAAGAAAAGGGGGGACTGGAAGGGCTAATTCACTC
CCAACGAAGACAAGATATCCTTGATCTGTGGATCTACCACACACAAGGCTACTTCCCTGATTGGCAGAACTACACACC
AGGGCCAGGGATCAGATATCCACTGACCTTTGGATGGTGCTACAAGCTAGTACCAGTTGAGCAAGAGAAGGTAGAAG
AAGCCAATGAAGGAGAGAACACCCGCTTGTTACACCCTGTGAGCCTGCATGGGATGGATGACCCGGAGAGAGAAGT
ATTAGAGTGGAGGTTTGACAGCCGCCTAGCATTTCATCACATGGCCCGAGAGCTGCATCCGGACTGTACTGGGTCTC
TCTGGTTAGACCAGATCTGAGCCTGGGAGCTCTCTGGCTAACTAGGGAACCCACTGCTTAAGCCTCAATAAAGCTTG
CCTTGAGTGCTTCAAGTAGTGTGTGCCCGTCTGTTGTGTGACTCTGGTAACTAGAGATCCCTCAGACCCTTTTAGTCA
GTGTGGAAAATCTCTAGCAGCATGTGAGCAAAAGGCCAGCAAAAGGCCAGGAACCGTAAAAAGGCCGCGTTGCTGG
CGTTTTTCCATAGGCTCCGCCCCCCTGACGAGCATCACAAAAATCGACGCTCAAGTCAGAGGTGGCGAAACCCGACA
GGACTATAAAGATACCAGGCGTTTCCCCCTGGAAGCTCCCTCGTGCGCTCTCCTGTTCCGACCCTGCCGCTTACCGG
ATACCTGTCCGCCTTTCTCCCTTCGGGAAGCGTGGCGCTTTCTCATAGCTCACGCTGTAGGTATCTCAGTTCGGTGTA
GGTCGTTCGCTCCAAGCTGGGCTGTGTGCACGAACCCCCCGTTCAGCCCGACCGCTGCGCCTTATCCGGTAACTAT
CGTCTTGAGTCCAACCCGGTAAGACACGACTTATCGCCACTGGCAGCAGCCACTGGTAACAGGATTAGCAGAGCGA
GGTATGTAGGCGGTGCTACAGAGTTCTTGAAGTGGTGGCCTAACTACGGCTACACTAGAAGAACAGTATTTGGTATC
TGCGCTCTGCTGAAGCCAGTTACCTTCGGAAAAAGAGTTGGTAGCTCTTGATCCGGCAAACAAACCACCGCTGGTAG
CGGTGGTTTTTTTGTTTGCAAGCAGCAGATTACGCGCAGAAAAAAAGGATCTCAAGAAGATCCTTTGATCTTTTCTAC
GGGGTCTGACGCTCAGTGGAACGAAAACTCACGTTAAGGGATTTTGGTCATGAGATTATCAAAAAGGATCTTCACCTA
GATCCTTTTAAATTAAAAATGAAGTTTTAAATCAATCTAAAGTATATATGAGTAAACTTGGTCTGACAGTTACCAATGCT
TAATCAGTGAGGCACCTATCTCAGCGATCTGTCTATTTCGTTCATCCATAGTTGCCTGACTCCCCGTCGTGTAGATAA
CTACGATACGGGAGGGCTTACCATCTGGCCCCAGTGCTGCAATGATACCGCGAGACCCACGCTCACCGGCTCCAGA
TTTATCAGCAATAAACCAGCCAGCCGGAAGGGCCGAGCGCAGAAGTGGTCCTGCAACTTTATCCGCCTCCATCCAGT
CTATTAATTGTTGCCGGGAAGCTAGAGTAAGTAGTTCGCCAGTTAATAGTTTGCGCAACGTTGTTGCCATTGCTACAG
GCATCGTGGTGTCACGCTCGTCGTTTGGTATGGCTTCATTCAGCTCCGGTTCCCAACGATCAAGGCGAGTTACATGA
TCCCCCATGTTGTGCAAAAAAGCGGTTAGCTCCTTCGGTCCTCCGATCGTTGTCAGAAGTAAGTTGGCCGCAGTGTT
ATCACTCATGGTTATGGCAGCACTGCATAATTCTCTTACTGTCATGCCATCCGTAAGATGCTTTTCTGTGACTGGTGA
GTACTCAACCAAGTCATTCTGAGAATAGTGTATGCGGCGACCGAGTTGCTCTTGCCCGGCGTCAATACGGGATAATA
CCGCGCCACATAGCAGAACTTTAAAAGTGCTCATCATTGGAAAACGTTCTTCGGGGCGAAAACTCTCAAGGATCTTAC
CGCTGTTGAGATCCAGTTCGATGTAACCCACTCGTGCACCCAACTGATCTTCAGCATCTTTTACTTTCACCAGCGTTT
CTGGGTGAGCAAAAACAGGAAGGCAAAATGCCGCAAAAAAGGGAATAAGGGCGACACGGAAATGTTGAATACTCATA
CTCTTCCTTTTTCAATATTATTGAAGCATTTATCAGGGTTATTGTCTCATGAGCGGATACATATTTGAATGTATTTAGAA
AAATAAACAAATAGGGGTTCCGCGCACATTTCCCCGAAAAGTGCCACCTGAC

> CDH26-Clover-N1 (partial sequence) 7232 bp CLOVER-fusion Infusion Primer Regions Clone Insert Sequence in Blue

CATGCATTAGTTATTAATAGTAATCAATTACGGGGTCATTAGTTCATAGCCCATATATGGAGTTCCGCGTTACATAACTTACGGTAAAT
GGCCCGCCTGGCTGACCGCCCAACGACCCCCGCCCATTGACGTCAATAATGACGTATGTTCCCATAGTAACGCCAATAGGGACTTTCC
ATTGACGTCAATGGGTGGAGTATTTACGGTAAACTGCCCACTTGGCAGTACATCAAGTGTATCATATGCCAAGTACGCCCCCTATTGA
CGTCAATGACGGTAAATGGCCCGCCTGGCATTATGCCCAGTACATGACCTTATGGGACTTTCCTACTTGGCAGTACATCTACGTATTA
GTCATCGCTATTACCATGGTGATGCGGTTTTGGCAGTACATCAATGGGCGTGGATAGCGGTTTGACTCACGGGGATTTCCAAGTCTCC
ACCCCATTGACGTCAATGGGAGTTTGTTTTGGCACCAAAATCAACGGGACTTTCCAAAATGTCGTAACAACTCCGCCCCATTGACGCA
AATGGGCGGTAGGCGTGTACGGTGGGAGGTCTATATAAGCAGAGCTGGTTTAGTGAACCGTCAGATCCGCTAGCGCTACCGGACTC
AGATCTCGAGCTCAAGCTTCGAATTCTGCAGTCGACGGTACCGCGGGCCCGGGATCATGGCCATGAGATCCGGGAGGCACCCCTCG
CTGCTGCTGCTTCTAGTGCTGCTGCTGTGGCTGCTGCAGGTCAGTATCATTGACAGTGTTCAACAGGAAACAGATGATCTTACTAAGC
AAACAAAGGAAAAGATCTACCAGCCTCTACGGCGATCCAAGAGAAGATGGGTTATCACCACCTTGGAGCTGGAGGAGGAAGACCCG
GGACCCTTTCCCAAACTCATTGGTGAGCTGTTCAATAATATGTCTTATAACATGTCACTAATGTATCTAATCAGTGGACCTGGTGTGGA
TGAATATCCAGAGATTGGTTTGTTTTCTCTAGAAGATCATGAGAACGGAAGGATATATGTTCACCGCCCTGTCGATCGAGAAATGACA
CCATCTTTCACGGTTTATTTTGATGTTGTGGAGCGCTCAACAGGAAAAATTGTGGATACATCCTTGATTTTCAACATTAGGATCAGTGA
TGTGAATGATCATGCACCCCAGTTTCCAGAGAAGGAATTTAACATCACTGTGCAAGAAAACCAATCTGCAGGGCAACCTATTTTTCAG
ATGTTAGCAGTCGATTTGGATGAAGAAAACACTCCAAATTCTCAAGTCCTTTACTTCCTCATTTCTCAAACACCATTACTGAAAGAAAG
TGGTTTCCGGGTTGATCGCCTTAGTGGAGAAATACGACTCTCTGGCTGCTTAGATTATGAGACCGCTCCTCAGTTTACACTGCTAATCA
GAGCCAGGGACTGTGGAGAACCGTCACTGTCATCCACGACCACCGTTCACGTGGATGTGCAAGAAGGCAACAACCACAGGCCTGCA
TTTACCCAGGAGAACTATAAGGTTCAGATTCCTGAAGGCCGAGCCAGCCAGGGCGTGTTGCGTCTCCTGGTTCAAGATCGAGATTCT
CCATTTACATCAGCTTGGAGAGCAAAATTCAACATATTGCATGGCAATGAAGAGGGGCATTTTGACATTTCGACTGACCCTGAGACCA
ACGAAGGGATATTAAATGTTATCAAGCCTTTGGATTATGAGACTCGCCCAGCGCAAAGCCTCATCATTGTCGTGGAGAATGAGGAGA
GGCTCGTCTTCTGTGAGAGAGGAAAGCTTCAGCCGCCAAGGAAGGCAGCAGCCAGCGCCACTGTGAGTGTGCAGGTGACAGACGC
CAACGACCCACCAGCCTTTCACCCCCAGAGCTTCATTGTCAATAAAGAGGAGGGCGCCAGGCCTGGGACCCTGTTGGGAACTTTTAA
TGCCATGGATCCAGACAGCCAGATAAGATATGAACTGGTTCATGACCCAGCAAATTGGGTCAGCGTCGACAAAAACTCCGGAGTGGT
CATCACCGTGGAGCCAATTGACCGAGAATCCCCTCATGTAAATAACAGTTTTTATGTAATCATCATTCACGCTGTTGATGATGGCTTCC
CACCGCAGACTGCTACAGGGACCCTAATGCTCTTCCTGTCTGACATCAATGACAACGTCCCGACTCTCCGGCCACGTTCCCGCTACAT
GGAGGTCTGTGAGTCTGCTGTGCATGAGCCCCTCCACATCGAGGCAGAGGATCCGGACCTGGAGCCGTTCTCTGACCCATTTACATTT
GAATTGGACAATACCTGGGGAAATGCGGAGGACACATGGAAGTTGGGGAGAAATTGGGGTCAATCAGTTGAACTTTTAACCTTGAG
AAGCCTGCCACGTGGTAATTACTTGGTGCCACTCTTCATTGGAGACAAACAGGGACTTTCCCAGAAGCAAACTGTCCATGTAAGGATC
TGCCCCTGTGCCAGTGGGCTCACATGTGTGGAGCTTGCAGATGCAGAAGTGGGGCTTCATGTGGGGGCCCTGTTCCCTGTCTGTGCA
GCATTTGTGGCTCTGGCAGTGGCTCTGCTTTTTCTGTTGCGATGCTATTTTGTGCTTGAACCTAAGAGGCATGGATGCTCTGTATCCAA
TGATGAAGGCCACCAAACACTGGTCATGTATAATGCGGAGAGCAAAGGCACTTCAGCCCAGACATGGTCAGATGTTGAAGGCCAGA
GGCCGGCTCTGCTCATCTGCACAGCTGCAGCAGGACCCACGCAGGGAGTTAAGGATCTCGAGGAAGTGCCTCCATCTGCAGCGAGT
CAGTCAGCCCAAGCACGCTGTGCTCTGGGGAGCTGGGGTTATGGCAAGCCCTTTGAGCCAAGAAGTGTGAAAAACATACACTCTACT
CCTGCTTACCCAGATGCCACAATGCACAGACAACTCCTGGCTCCGGTGGAAGGAAGGATGGCAGAGACATTGAATCAGAAACTCCAT
GTTGCCAATGTGCTGGAAGATGACCCCGGCTACCTACCTCACGTCTACAGCGAGGAAGGGGAGTGTGGAGGGGCCCCATCCCTCAG
CTCTCTGGCCAGCTTGGAACAGGAGTTGCAACCTGATTTGCTGGACTCTTTGGGTTCAAAAGCGACTCCGTTTGAGGAAATATATTCA
GAGTCAGGTGTTCCTTCCGATCCACCGGTCGCCACCATGGTGAGCAAGGGCGAGGAGCTGTTCACCGGGGTGGTGCCCATCCTGGTC
GAGCTGGACGGCGACGTAAACGGCCACAAGTTCAGCGTCCGCGGCGAGGGCGAGGGCGATGCCACCAACGGCAAGCTGACCCTGA
AGTTCATCTGCACCACCGGCAAGCTGCCCGTGCCCTGGCCCACCCTCGTGACCACCTTCGGCTACGGCGTGGCCTGCTTCAGCCGCTA
CCCCGACCACATGAAGCAGCACGACTTCTTCAAGTCCGCCATGCCCGAAGGCTACGTCCAGGAGCGCACCATCTCTTTCAAGGACGA
CGGTACCTACAAGACCCGCGCCGAGGTGAAGTTCGAGGGCGACACCCTGGTGAACCGCATCGAGCTGAAGGGCATCGACTTCAAGG
AGGACGGCAACATCCTGGGGCACAAGCTGGAGTACAACTTCAACAGCCACTACGTCTATATCACGGCCGACAAGCAGAAGAACAGC
ATCAAGGCTAACTTCAAGATCCGCCACAACGTTGAGGACGGCAGCGTGCAGCTCGCCGACCACTACCAGCAGAACACCCCCATCGGC
GACGGCCCCGTGCTGCTGCCCGACAACCACTACCTGAGCCATCAGTCCAAGCTGAGCAAAGACCCCAACGAGAAGCGCGATCACATG
GTCCTGCTGGAGTTCGTGACCGCCGCCGGGATCACTCTCGGCATGGACGAGCTGTACAAGTAAGCGGCCGCGACTCTAGATCATAAT

CAGCCATACCACATTTGTAGAGGTTTTACTTGCTTTAAAAAACCTCCCACACCTCCCCCTGAACCTGAAACATAAAATGAATGCAATTG
TTGTTGTTAACTTGTTTATTGCAGCTTATAATGGTTACAAATAAAGCAATAGCATCACAAATTTCACAAATAAAGCATTTTTTTCACTGC
ATTCTAGTTGTGGTTTGTCCAAACTCATCAATGTATCTTAAGGCGTAAATTGTAAGCGTTAATATTTTGTTAAAATTCGCGTTAAATTTT
TGTTAAATCAGCTCATTTTTTAACCAATAGGCCGAAATCGGCAAAATCCCTTATAAATCAAAAGAATAGACCGAGATAGGGTTGAGTG
TTGTTCCAGTTTGGAACAAGAGTCCACTATTAAAGAACGTGGACTCCAACGTCAAAGGGCGAAAAACCGTCTATCAGGGCGATGGCC
CACTACGTGAACCATCACCCTAATCAAGTTTTTTGGGGTCGAGGTGCCGTAAAGCACTAAATCGGAACCCTAAAGGGAGCCCCCGAT
TTAGAGCTTGACGGGGAAAGCCGGCGAACGTGGCGAGAAAGGAAGGGAAGAAAGCGAAAGGAGCGGGCGCTAGGGCGCTGGCA
AGTGTAGCGGTCACGCTGCGCGTAACCACCACACCCGCCGCGCTTAATGCGCCGCTACAGGGCGCGTCAGGTGGCACTTTTCGGGG
AAATGTGCGCGGAACCCCTATTTGTTTATTTTTCTAAATACATTCAAATATGTATCCGCTCATGAGACAATAACCCTGATAAATGCTTC
AATAATATTGAAAAAGGAAGAGTCCTGAGGCGGAAAGAACCAGCTGTGGAATGTGTGTCAGTTAGGGTGTGGAAAGTCCCCAGGCT
CCCCAGCAGGCAGAAGTATGCAAAGCATGCATCTCAATTAGTCAGCAACCAGGTGTGGAAAGTCCCCAGGCTCCCCAGCAGGCAGA
AGTATGCAAAGCATGCATCTCAATTAGTCAGCAACCATAGTCCCGCCCCTAACTCCGCCCATCCCGCCCCTAACTCCGCCCAGTTCCGC
CCATTCTCCGCCCCATGGCTGACTAATTTTTTTTATTTATGCAGAGGCCGAGGCCGCCTCGGCCTCTGAGCTATTCCAGAAGTAGTGAG
GAGGCTTTTTTGGAGGCCTAGGCTTTTGCAAAGATCGATCAAGAGACAGGATGAGGATCGTTTCGCATGATTGAACAAGATGGATTG
CACGCAGGTTCTCCGGCCGCTTGGGTGGAGAGGCTATTCGGCTATGACTGGGCACAACAGACAATCGGCTGCTCTGATGCCGCCGTG
TTCCGGCTGTCAGCGCAGGGGCGCCCGGTTCTTTTTGTCAAGACCGACCTGTCCGGTGCCCTGAATGAACTGCAAGACGAGGCAGCG
CGGCTATCGTGGCTGGCCACGACGGGCGTTCCTTGCGCAGCTGTGCTCGACGTTGTCACTGAAGCGGGAAGGGACTGGCTGCTATT
GGGCGAAGTGCCGGGGCAGGATCTCCTGTCATCTCACCTTGCTCCTGCCGAGAAAGTATCCATCATGGCTGATGCAATGCGGCGGCT
GCATACGCTTGATCCGGCTACCTGCCCATTCGACCACCAAGCGAAACATCGCATCGAGCGAGCACGTACTCGGATGGAAGCCGGTCT
TGTCGATCAGGATGATCTGGACGAAGAGCATCAGGGGCTCGCGCCAGCCGAACTGTTCGCCAGGCTCAAGGCGAGCATGCCCGACG
GCGAGGATCTCGTCGTGACCCATGGCGATGCCTGCTTGCCGAATATCATGGTGGAAAATGGCCGCTTTTCTGGATTCATCGACTGTG
GCCGGCTGGGTGTGGCGGACCGCTATCAGGACATAGCGTTGGCTACCCGTGATATTGCTGAAGAGCTTGGCGGCGAATGGGCTGAC
CGCTTCCTCGTGCTTTACGGTATCGCCGCTCCCGATTCGCAGCGCATCGCCTTCTATCGCCTTCTTGACGAGTTCTTCTGAGCGGGACT
CTGGGGTTCGAAATGACCGACCAAGCGACGCCCAACCTGCCATCACGAGATTTCGATTCCACCGCCGCCTTCTATGAAAGGTTGGGC
TTCGGAATCGTTTTCCGGGACGCCGGCTGGATGATCCTCCAGCGCGGGGATCTCATGCTGGAGTTCTTCGCCCACCCTAGGGGGAGG
CTAACTGAAACACGGAAGGAGACAATACCGGAAGGAACCCGCGCTATGACGGCAATAAAAAGACAGAATAAAACGCACGGTGTTG
GGTCGTTTGTTCATAAACGCGGGGTTCGGTCCCAGGGCTGGCACTCTGTCGATACCCCACCGAGACCCCATTGGGGCCAATACGCCC
GCGTTTCTTCCTTTTCCCCACCCCACCCCCCAAGTTCGGGTGAAGGCCCAGGGCTCGCAGCCAACGTCGGGGCGGCAGGCCCTGCCAT
AGCCTCAGGTTACTCATATATACTTTAGATTGATTTAAAACTTCATTTTTAATTTAAAAGGATCTAGGTGAAGATCCTTTTTGATAATCT
CATGACCAAAATCCCTTAACGTGAGTTTTCGTTCCACTGAGCGTCAGACCCCGTAGAAAAGATCAAAGGATCTTCTTGAGATCCTTTTT
TTCTGCGCGTAATCTGCTGCTTGCAAACAAAAAAACCACCGCTACCAGCGGTGGTTTGTTTGCCGGATCAAGAGCTACCAACTCTTTTT
CCGAAGGTAACTGGCTTCAGCAGAGCGCAGATACCAAATACTGTTCTTCTAGTGTAGCCGTAGTTAGGCCACCACTTCAAGAACTCTG
TAGCACCGCCTACATACCTCGCTCTGCTAATCCTGTTACCAGTGGCTGCTGCCAGTGGCGATAAGTCGTGTCTTACCGGGTTGGACTC
AAGACGATAGTTACCGGATAAGGCGCAGCGGTCGGGCTGAACGGGGGGTTCGTGCACACAGCCCAGCTTGGAGCGAACGACCTAC
ACCGAACTGAGATACCTACAGCGTGAGCTATGAGAAAGCGCCACGCTTCCCGAAGGGAGAAAGGCGGACAGGTATCCGGTAAGCG
GCAGGGTCGGAACAGGAGAGCGCACGAGGGAGCTTCCAGGGGGAAACGCCTGGTATCTTTATAGTCCTGTCGGGTTTCGCCACCTC
TGACTTGAGCGTCGATTTTTGTGATGCTCGTCAGGGGGGCGGAGCCTATGGAAAAACGCCAGCAACGCGGCCTTTTTACGGTTCCTG
GCCTTTTGCTGGCCTTTTGCTCACATGTTCTTTCCTGCGTTATCCCCTGATTCTGTGGATAACCGTATTACCGC

Figure S9: qPCR Primers for Planar Cell Polarity Proteins.

Description	Name	Oligo Sequence
Forward RT	DVL1	CTCCTCCTTCAGCAGCATAACC
Reverse RT	DVL1	TCATGATGGAGCCAATGTAGATG
TaqMan Fwd	DVL1	CTCCACCATGTCCCTCAACA
TaqMan Rvs	DVL1	ATGCTGATGCCCAGAAAGTG
TaqMan Probe	DVL1	TCGTCACTGTCACGCTCAACATGGA-FAM(BHQ)
Forward RT	PRICKLE2	GCCCTACCATTATGGGAACAAGAT
Reverse RT	PRICKLE2	CCTGGACTGTAGGAAGTTCTGATG
TaqMan Fwd	PRICKLE2	TGGGAACAAGATGGAGCAGA
TaqMan Rvs	PRICKLE2	GATGTTGCACTGGCTGAGGA
TaqMan Probe	PRICKLE2	CCAGACCCAGAGCCCTCTGCAGC-FAM(BHQ)
Forward RT	VANG1	AACTTGGAGCCACTTAATTTCCTG
Reverse RT	VANG1	AGCCTCTTTCCTGGTCCTAAGTG
TaqMan Fwd	VANG1	CCTGCCAAAGGAGACCAAAC
TaqMan Rvs	VANG1	AACACAGGGACTGGACAGCA
TaqMan Probe	VANG1	ATGCCAACCACGCTGGCAACTG-FAM(BHQ)
Forward RT	CRB3	CCACTTCTGCAAATGAGAATAGCA
Reverse RT	CRB3	AGGAGCAAGGCAGCCAAGAG
TaqMan Fwd	CRB3	CCTTCATCCACCAGCTCCA
TaqMan Rvs	CRB3	GCAGCCAAGAGGGAGAAGAC
TaqMan Probe	CRB3	TTCTGGACGCAGGTTGCCATCG-FAM(BHQ)
Forward RT	CLSR3	GTCGCTACCCTCGCTACCA
Reverse RT	CLSR3	CTTGTGGGCAGAACTTCAGATG
TaqMan Fwd	CLSR3	CCCTCGCTACCATAGCAACC
TaqMan Rvs	CLSR3	CAGCACATGGGTGTGAGGAT
TaqMan Probe	CLSR3	CCAGGCATCCTGGCCTCGAAAG-FAM(BHQ)
Forward RT	CETN2	TGATGATGATGAAACTGGGAAGAT
Reverse RT	CETN2	AGGAACTCTTGCTCACTGACCTCT
TaqMan Fwd	CETN2	GCCAAGGAGTTGGGTGAGA
TaqMan Rvs	CETN2	CCATCTCCATCTCGATCAGC
TaqMan Probe	CETN2	TTTCCTGCAGCTCCTCATCAGTCAGGT-FAM(BHQ)
Forward RT	EEF1A1	TGCTAACATGCCTTGGTTCAAG
Reverse RT	EEF1A1	TTGGACGAGTTGGTGGTAGGAT
TaqMan Fwd	EEF1A1	CCTTGGTTCAAGGGATGGAA
TaqMan Rvs	EEF1A1	GCCTCAAGCAGCGTGGTT
TaqMan Probe	EEF1A1	CACTGGCATTGCCATCCTTACGGG-FAM(BHQ)
Forward RT	RPL13A	GGACCGTGCGAGGTATGCT
Reverse RT	RPL13A	TTCAGACGCACGACCTTGAG
TaqMan Fwd	RPL13A	TATGCTGCCCCACAAAACC
TaqMan Rvs	RPL13A	TGCCGTCAAACACCTTGAGA
TaqMan Probe	RPL13A	CAGAGCGGCCTGGCCTCGCT-FAM(BHQ)
Forward RT	PPIA	ATGAGAACTTCATCCTAAAGCATACG
Reverse RT	PPIA	TTGGCAGTGCAGATGAAAAACT
TaqMan Fwd	PPIA	ACGGGTCCTGGCATCTTGT
TaqMan Rvs	PPIA	GCAGATGAAAAACTGGGAACCA
TaqMan Probe	PPIA	ATGGCAAATGCTGGACCCAACACA-FAM(BHQ)
